# Genomic Targets of Brachyury (T) in Differentiating Mouse Embryonic Stem Cells

**DOI:** 10.1371/journal.pone.0033346

**Published:** 2012-03-30

**Authors:** Amanda L. Evans, Tiago Faial, Michael J. Gilchrist, Thomas Down, Ludovic Vallier, Roger A. Pedersen, Fiona C. Wardle, James C. Smith

**Affiliations:** 1 Wellcome Trust/Cancer Research UK Gurdon Institute, University of Cambridge, Cambridge, United Kingdom; 2 The Anne McLaren Laboratory for Regenerative Medicine, University of Cambridge, Cambridge, United Kingdom; 3 Department of Zoology, University of Cambridge, Cambridge, United Kingdom; 4 Medical Research Council, National Institute for Medical Research, London, United Kingdome; 5 Randall Division of Cell and Molecular Biophysics, King's College London, London, United Kingdom; Brigham and Women's Hospital, United States of America

## Abstract

**Background:**

The T-box transcription factor Brachyury (T) is essential for formation of the posterior mesoderm and the notochord in vertebrate embryos. Work in the frog and the zebrafish has identified some direct genomic targets of Brachyury, but little is known about Brachyury targets in the mouse.

**Methodology/Principal Findings:**

Here we use chromatin immunoprecipitation and mouse promoter microarrays to identify targets of Brachyury in embryoid bodies formed from differentiating mouse ES cells. The targets we identify are enriched for sequence-specific DNA binding proteins and include components of signal transduction pathways that direct cell fate in the primitive streak and tailbud of the early embryo. Expression of some of these targets, such as *Axin2, Fgf8* and *Wnt3a,* is down regulated in *Brachyury* mutant embryos and we demonstrate that they are also Brachyury targets in the human. Surprisingly, we do not observe enrichment of the canonical T-domain DNA binding sequence 5′-TCACACCT-3′ in the vicinity of most Brachyury target genes. Rather, we have identified an (AC)_n_ repeat sequence, which is conserved in the rat but not in human, zebrafish or *Xenopus*. We do not understand the significance of this sequence, but speculate that it enhances transcription factor binding in the regulatory regions of Brachyury target genes in rodents.

**Conclusions/Significance:**

Our work identifies the genomic targets of a key regulator of mesoderm formation in the early mouse embryo, thereby providing insights into the Brachyury-driven genetic regulatory network and allowing us to compare the function of Brachyury in different species.

## Introduction

*Brachyury* (*T*) is expressed in the primitive streak, tailbud and notochord of the early mouse embryo [1], [2]. It plays a key role in early development: mouse embryos lacking functional Brachyury protein do not gastrulate properly, fail to form a differentiated notochord, lack structures posterior to somite seven, and have defects in left-right patterning [3], [4], [5]. The expression patterns of the *Xenopus* [6] and zebrafish [7], [8] *Brachyury* orthologues resemble those of the mouse, and these genes play similar roles in early development [8], [9], [10], [11], indicating that *Brachyury* function has been conserved throughout evolution.

In an effort to understand how Brachyury exerts its effects, we have searched for genomic targets of this transcription factor. In previous work using *Xenopus* embryos we have used differential screening approaches to isolate target genes such as *eFGF* [12], members of the *Bix* family [13], [14] and *Wnt11* [15], while a chromatin immunoprecipitation-microarray (ChIP-chip) approach in the zebrafish embryo has allowed us to identify more than 200 potential targets of No-tail a (Ntla), the orthologue of Brachyury [16]. In this paper, we apply a ChIP-chip approach to identify targets of Brachyury during mouse embryonic stem (ES) cell differentiation. ES cells provide an abundant source of material as they differentiate towards embryoid bodies (EBs), and we predict that the identification of Brachyury targets in these cells will shed light on ES cell differentiation as well as help identify such targets in the early embryo. This work might also indicate the extent to which the biological function of Brachyury has been conserved in vertebrates and provide information on how Brachyury binding motifs are disposed within target cis-regulatory regions.

Our results show that Brachyury targets in differentiating ES cells are enriched for sequence-specific DNA binding proteins and components of signal transduction pathways that direct cell fate in the primitive streak and tailbud of the early embryo. Interestingly, most binding peaks were not enriched for the canonical T-box binding site 5′-TCACACCT-3′ [17], [18] but did contain a repeating AC motif. Amongst the signal transduction pathway components were regulators of the WNT and FGF pathways. These include *Axin2* (*Axil*/*Conductin*), which encodes a negative regulator of Wnt signalling, as well as *Wnt3a* and *Fgf8*. Significantly, expression of all three genes is down regulated in homozygous *Brachyury* mutant embryos and we show by ChIP-qPCR that these are also genomic targets of BRACHYURY in differentiating human ES cells. These results are consistent with work in the zebrafish emphasising the importance of Wnt and Fgf signal transduction pathway components as Brachyury targets [16]. In demonstrating that expression of *Axin2* in the early mouse embryo is regulated by Brachyury as well as by TCF/Lef proteins [19], [20], [21], [22], our results emphasise the complex interplay between signalling pathways in the regulation of gene expression in the early embryo.

## Results

### ES cell culture

**Figure 1:**
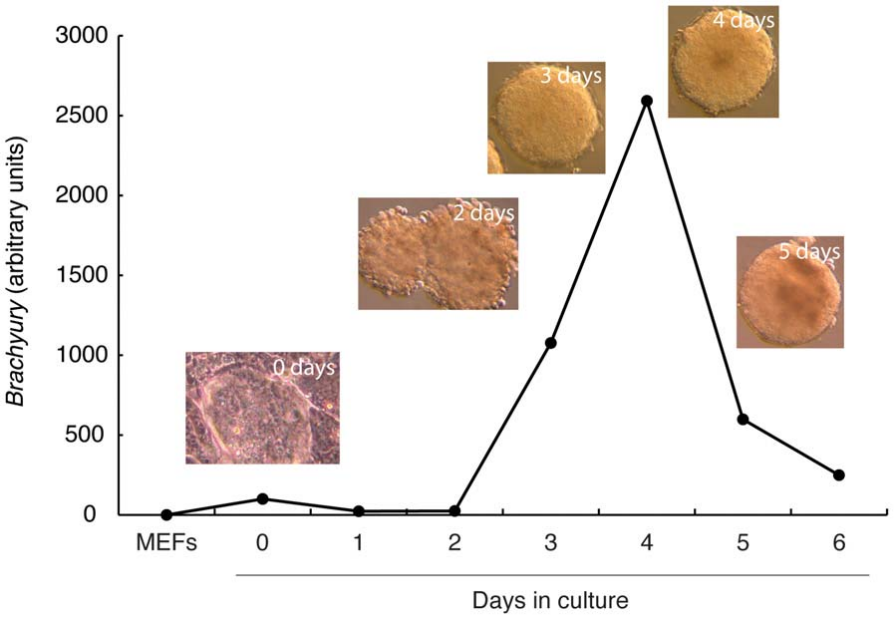
Temporal expression pattern of *Brachyury* during early ES cell differentiation. The graph shows a quantitative RT-PCR profile from an embryoid body spinner culture. *Brachyury* expression is calculated relative to *beta actin*. Images show undifferentiated R1 cells on mouse embryo fibroblast feeders at day 0, early blast colonies at day 2, and embryoid bodies at days 3, 4 (when they are cross-linked) and 5.

Preliminary experiments demonstrated that our ES cell culture regime yielded embryoid bodies of uniform size, similar to that of EBs grown on hydrophobic surfaces [23], and that expression of *Brachyury* usually peaked at day 4 of differentiation (Fig. 1). Immunohistochemical analysis indicated that approximately 15% of cells in our embryoid bodies express Brachyury (data not shown).

### ChIP-chip and bioinformatic analysis

Binding sites of Brachyury were identified by ChIP-chip experiments and the closest gene was identified using version NBI35.1 of the annotated mouse genome (see Material and Methods). Following filtration, our analysis gave a list of 520 enriched probes representing 396 genes (Table S1). Genomic quantitative PCR on a selection of genes called as bound or unbound confirmed that our ChIP-chip approach identified genuine binding events (Fig. S1).

**Figure 2:**
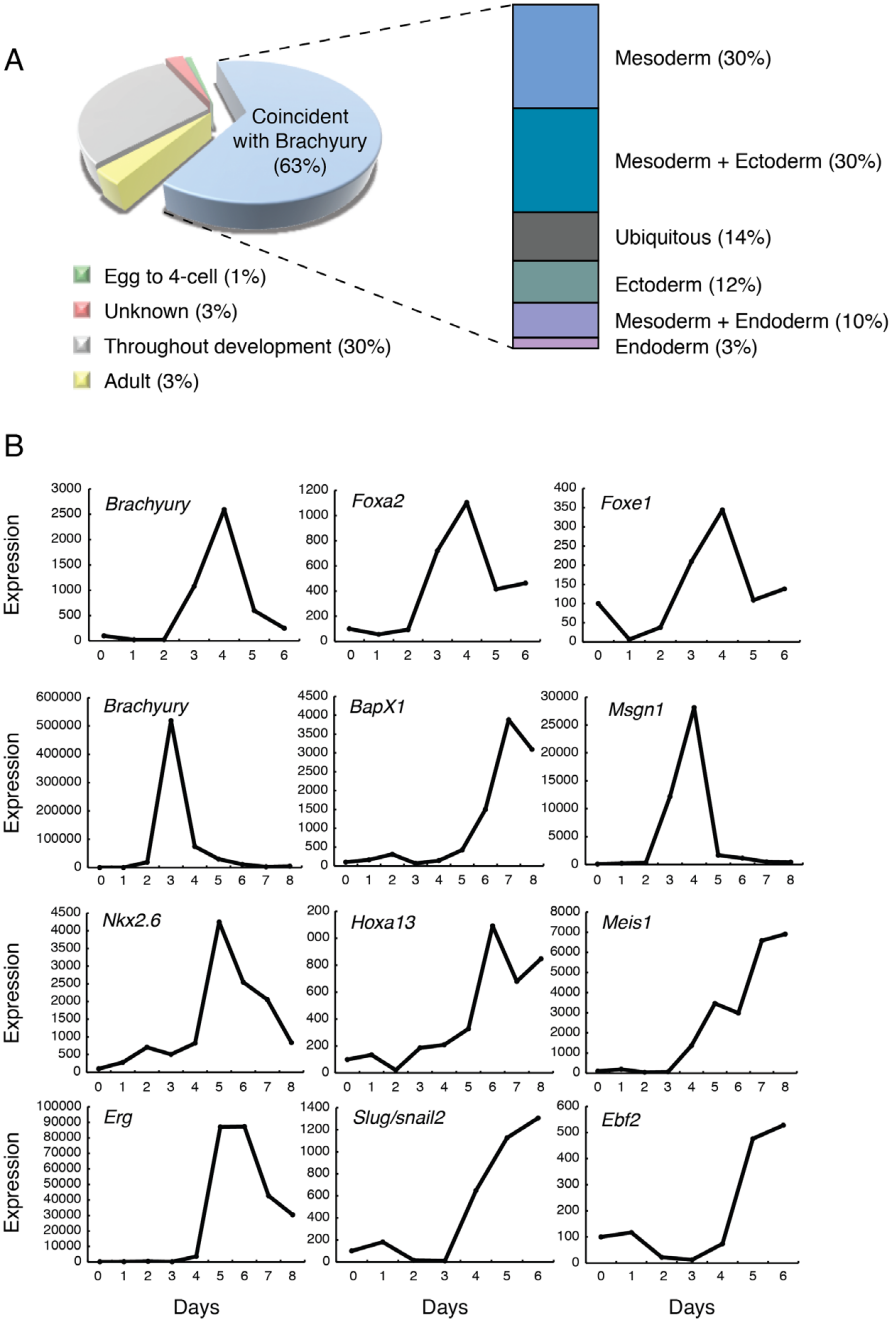
Analysis of Brachyury targets. (A) Pie chart showing the times in development at which Brachyury target gene expression begins in the mouse embryo (as a percentage of total; n = 396). Most genes (63%) start to be expressed between E7.5, when *Brachyury* is expressed in the primitive streak, notochord and tailbud, and E17.5, when expression is restricted to trunk mesenchyme. Of targets showing this temporal expression pattern, 30% are restricted to mesodermal derivatives, as indicated in the bar chart to the right. Others are expressed in various combinations of ectoderm, mesoderm and endoderm. (B) Temporal expression of transcription factor targets of Brachyury during ES cell differentiation in spinner culture, obtained by RT-PCR. The three panels in the top row were taken from a batch of cells in which *Brachyury* expression peaked at day 4 of culture; the rest were taken from a batch in which *Brachyury* expression peaked at day 3. All show means of triplicate measurements and are normalised to levels of *beta actin*. *Foxa2* and *Foxe1* in the top row peak with *Brachyury* at day 4; genes in the lower panel peak later than *Brachyury*.

*Brachyury* is expressed at its highest levels in the primitive streak and haematopoietic progenitors at E7.5 to E8.5. Later expression is restricted to the tailbud and notochord (E12.5), and then to parts of the brain and tail [2]. Of the Brachyury targets identified in our embryoid body experiments whose expression patterns are known, most (63%) are activated during this period of 7.5 to 17.5 dpc of mouse development (Fig. 2A). And of these 250 genes, many are restricted to the primitive streak or its mesodermal derivatives, with 30% (75 transcripts) expressed exclusively in the mesoderm (Fig. 2A). In addition to *Axin2*, *Wnt3a* and *Fgf8*, which are discussed below, genes that have been reported to be co-expressed with *Brachyury* include *Msgn1*, whose expression is down regulated in *Brachyury* mutants [24], *Meis1* [25] *Trim 28* [26] and *Zic2* [27], which are expressed in the primitive streak during gastrulation, *Foxa2*, present in the node and notochord [28], and *Adam19* (*meltrin beta*), present in tailbud mesenchyme [29]. These and other transcripts (see below) are also co-expressed with *Brachyury* (or are activated shortly after *Brachyury*) in embryoid bodies, and typical profiles of *Msgn1, Meis1* and *Foxa2* expression are shown in Fig. 2B.

The targets we identify include components of the WNT, MAPK, JNK, TGF-β, Hedgehog, FGF and G-protein coupled signal transduction pathways (Table S2). Analysis of the targets yielded a set of gene ontology (GO) terms consistent with the function of *Brachyury* during gastrulation [30].

In particular, cellular component analysis highlights gene products involved in morphogenesis, cell adhesion and cell polarity. Targets such as *Gdf5*, *Lmx1b* and *Dlx5* are involved in morphogenesis (Fig. S2A: GO:0048598; P<10^−6^), *Gabra1*, *Gfra3* and *Cbln3* are involved in anchoring the plasma membrane to cytoskeletal proteins (Fig. S2B: GO:0030054; P = 1×10^−3^) and others encode proteins involved in cell adhesion, such as the glycosyltransferases *B4galnt2* and *Cml4*/*Nat8*. This last observation is consistent with data showing that glycosyltransferases, and especially galactotransferases, are mis-expressed in T/T mutant mice [31], that the extracellular matrix is reduced in such embryos [32], and that cells have fewer cytoplasmic processes, especially in the somites and mesenchyme [32]. Significantly, over-expression of Cml4, or its *Xenopus* orthologue *Camello* (*Xcml*), inhibits gastrulation in *Xenopus* [33].

Interestingly, another group of targets is associated with germ cells (Table S3), including *Asap1* (*Ddef1*), which encodes an ADP-ribosylation factor GTPase-activating protein implicated in metastatic prostate cancer [34], and also the Wilms' tumour gene *WT1* [35].

### Transcription factor targets

Of the 396 genes identified as potential targets of Brachyury, 53 (13.4%) are transcription factors (Table S4), and indeed gene ontology analysis demonstrates significant enrichment for sequence-specific DNA binding proteins (Fig. S2C GO:0043565, p<10^−5^). Several families of transcription factors are represented, including the Ets, paired box, homeobox, winged helix/forkhead, bZip and zinc finger families. Under our ES cell culture conditions, expression of many of these transcription factors peaks either at the same time as *Brachyury* (*Foxa2*, *Foxe1*) or just afterwards (*BapX1*, *Ebf2*, *Erg*, *Hoxa13*, *Meis1*, *Msgn1*, *Nkx2.6* and *Slug*) (Fig. 2B). As we discuss below, our data provide a basis for deciphering the transcription factor genetic regulatory network underlying mesoderm formation in ES cells and in the embryo.

### T-box protein binding motifs

Previous work indicates that Brachyury interacts with the sequence 5′-TCACACCT-3′ [16], [36], [37], [38]. To our surprise, neither nested MICA nor RSAT identified this motif as significantly enriched in the DNA sequences selected in our experiments. Rather, both packages identified enrichment of the simple sequence repeat (AC)_n_ (Fig. S3A). However, although it was not enriched, we did observe that several regulatory regions contain a sequence resembling a T-box site (in which 1 to 3 nucleotides differ from the consensus) close to an AC repeat. These genes include *Axin2*, *Ctnnb1/β-catenin*, *Erg*, *Etv1, Fgf8*, *Fev*, *Foxa2*, *Foxe1*, *Fyb*, *Id4*, *Meis1,* and *Hoxa3* where the T-box like sites may be positioned either 5′ or 3′ to the repeat sequence.

To assess the significance of these observations we first performed electrophoretic mobility shift assays (Fig. S3B). As expected, the T-domain of mouse Brachyury binds the canonical TCACACCT sequence, binding can be competed by unlabelled oligonucleotide, and the complex can be ‘super-shifted’ by a Brachyury antibody. The (AC)_n_ repeat motif also forms a complex with Brachyury, but although the complex can also be ‘supershifted’, unlabelled oligonucleotide competes very poorly. Finally, when both motifs are present in the radiolabelled oligonucleotide, competition using an excess of cold oligonucleotide in which just the T-box site is mutated is poor, and so is competition in which just the (AC)_n_ region is mutated. Together, these observations indicate that the (AC)_n_ sequence interacts only weakly with Brachyury, if at all, and that its role may be restricted to stabilizing binding to an adjacent or even a distant T box site.

If true, such a role is likely to be restricted to rodent species. Our dataset contains 111 peaks with associated AC repeats longer than eight nucleotides (Table S5). Comparison with rat, human, zebrafish and *Xenopus* genomes shows that 38 of these AC-rich regions are unique to the mouse while 68 are also present in the rat. Sixteen of the AC repeats are present in the human genome, of which 11 are also present in rat. However, none of the repeats are conserved in zebrafish or *Xenopus* (Table S5).

### *Axin2* and *Wnt3a* as targets of Brachyury

**Figure 3:**
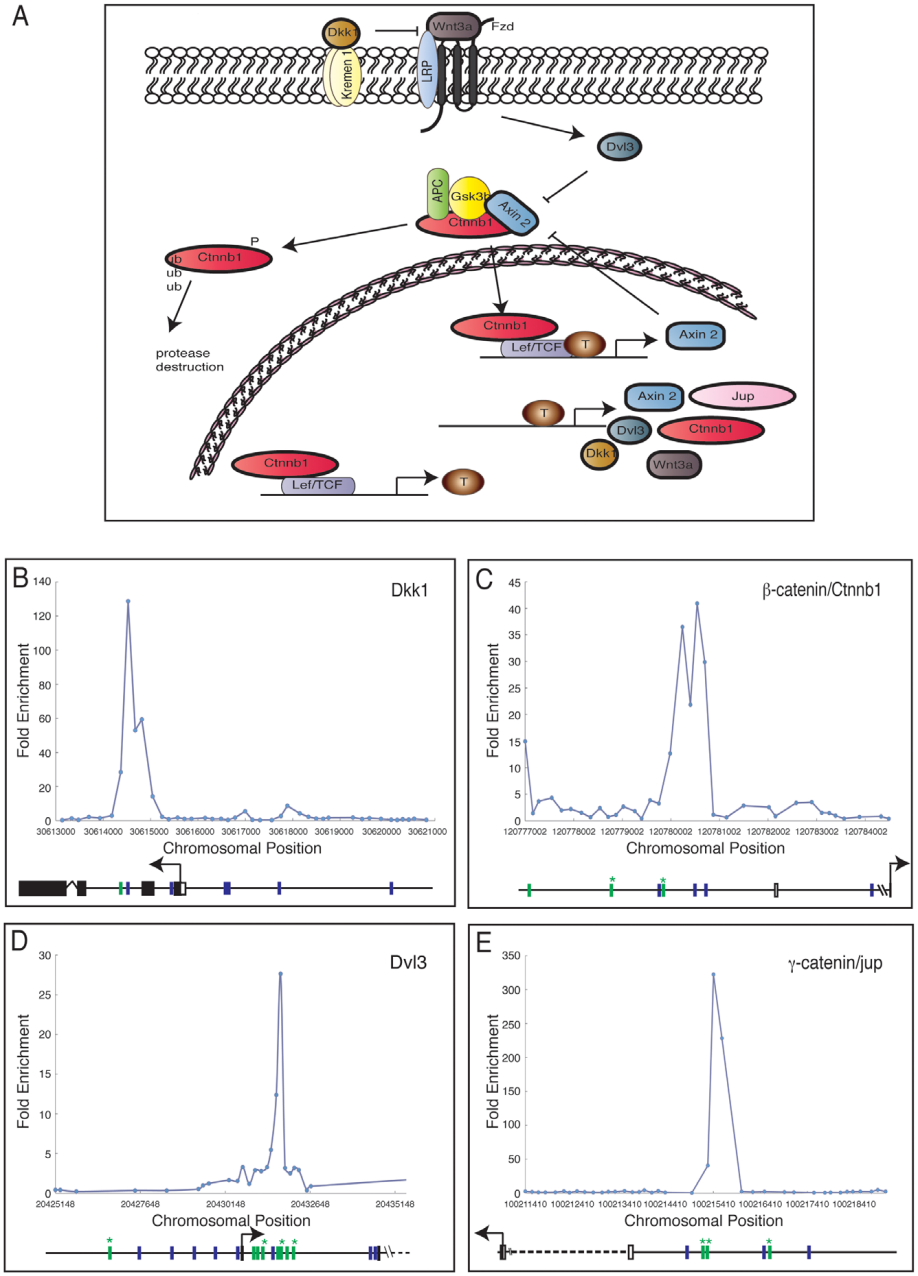
Components of the Wnt pathway as Brachyury targets. (A) The Wnt signalling pathway. Arrows indicate positive interactions and bars represent negative interactions. Targets identified in this study are outlined in **bold**. (B–E) Brachyury binding in genomic regions around *Dkk1* (B); *Ctnnb1*/*β-catenin* (C); *Dvl3* (D); and *γ-Catenin*/*jup*/*plakoglobin* (E). Each target shows fold enrichment against chromosomal position. Blue bars represent the T box-like site TSACANNT (N = any base, S = G/C) and green bars represent (AC)_n_. Stars above bars represent sequence on the reverse strand. Plots are average of triplicate chip results, aligned to the mm8 Feb. 2006 assembly.

**Figure 4:**
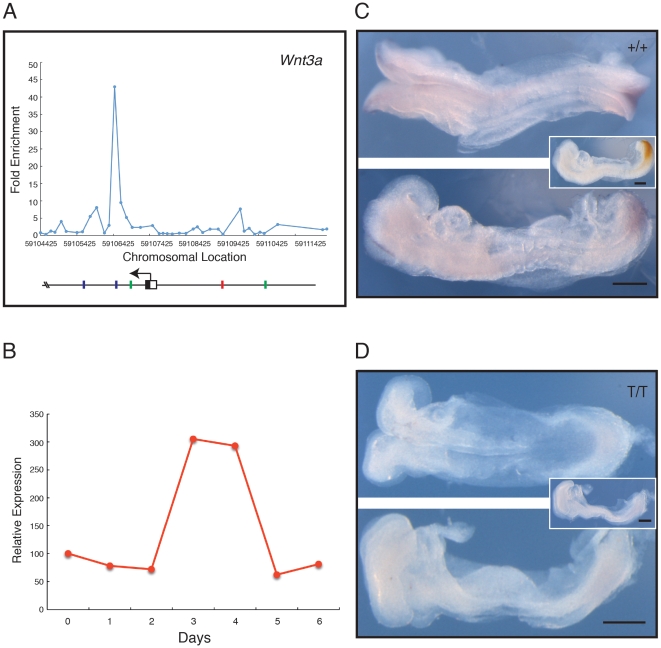
Analysis of *Wnt3a*, a positive regulator of the Wnt pathway. (A) Location analysis of *Wnt3a*. The figure (and Figs. 5, 6) shows fold enrichment against chromosomal position. Plot is the mean of triplicate chip results, aligned to the mm8 Feb. 2006 assembly. Blue bars represent the T box-like site TSACANNT (N = any base, S = G/C); green bars represent (AC)_n_; red bars the consensus TCACACCT. Stars above bars represent sequence on reverse strand. (B) Quantitative RT-PCR expression profile for *Wnt3a* during ES cell differentiation, expressed relative to *beta actin*. (C, D) Expression of *Wnt3a* studied by in situ hybridisation; in each, the top image shows a dorsal view, and the bottom image a lateral view. (C) Phenotypically wild type (+/+ or +/*T*) embryo at E8.5–8.75, and (D) a mutant (*T*/*T*) embryo from crosses of *Brachyury* heterozygous mutant mice. *Wnt3a* expression is detected with NBT/BCIP (purple) and the insets show a lateral view after double staining for *Brachyury* detected with INT/BCIP (orange brown). Note that in the wild type embryo *Wnt3a* is expressed in tailbud and paraxial mesoderm. In the mutant embryo expression of *Wnt3a* staining is absent or greatly reduced (n = 3). Scale bars indicate 250 µm.

**Figure 5:**
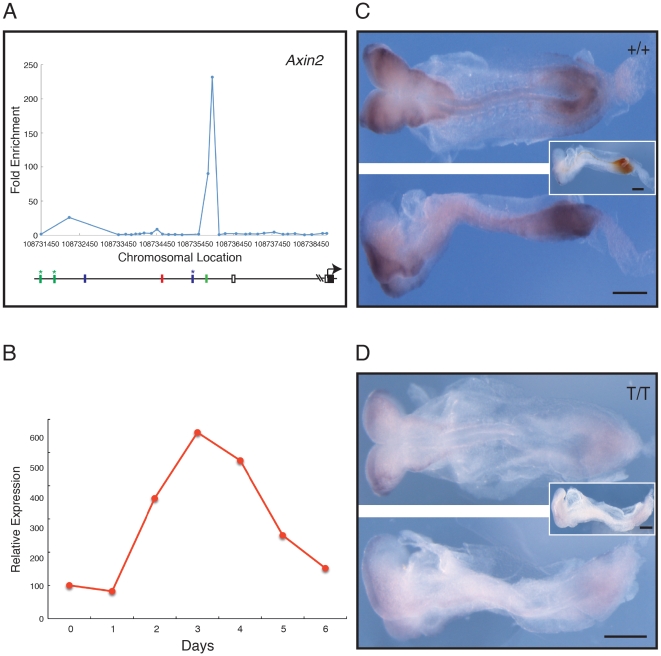
Analysis of *Axin2*, a negative regulator of the Wnt pathway. (A) Location analysis of *Axin2*. For details see legend to Fig. 4. (B) Quantitative RT-PCR expression profile for *Axin2* during ES cell differentiation, expressed relative to *beta actin*. (C, D) Expression of *Axin2* studied by in situ hybridisation; in each, the top image shows a dorsal view, and the bottom image a lateral view. (C) Phenotypically wild type (+/+ or +/*T*) embryo at E8.5–8.75 and (D) a mutant (*T*/*T*) embryo, both derived from crosses of *Brachyury* heterozygous mutant mice. *Axin2* expression is detected with NBT/BCIP (purple) and the insets show a lateral view after double staining for *Brachyury* detected with INT/BCIP (orange brown). Note that in the wild type embryo *Axin2* is expressed in tailbud, paraxial mesoderm and lateral margin of the neural folds. In the mutant embryo expression of *Axin2* is greatly reduced (n = 9). Scale bars are 250 µm.

Amongst the identified Brachyury targets are many genes encoding positive and negative regulators of the Wnt signalling pathway (Fig. 3A). Enrichment peaks in the promoter regions of *Dkk1*, *Ctnnb1*/β*-catenin*, *Dvl3*, and γ-*catenin*/*Jup* show Brachyury binding (Fig. 3B, C, D and E) and also reveal the presence of AC repeats (green bars) and imperfect T-binding sites (blue bars). Of these Wnt-related genes, *Wnt3a* and *Axin2* both show strong Brachyury binding peaks around their transcription start sites in our ChIP-chip analyses (Figs. 4A, 5A), and their temporal expression patterns both resemble that of *Brachyury* in our embryoid body system (Figs. 4B, 5B). For *Wnt3a*, a variant Brachyury site is positioned close to an AC repeat sequence in the first intron, and a canonical TCACACCT Brachyury site is upstream of the transcription start site (Fig. 4A). In the case of *Axin2*, a canonical Brachyury site is positioned close to a variant site and to an AC repeat (Fig. 5A).

To ask whether Brachyury is required for expression of *Wnt3a* and *Axin2*, we crossed mice that are heterozygous for a *Brachyury* mutation [39] and assessed expression of the two genes. In wild-type embryos at E7.5 the expression patterns of *Brachyury*, *Wnt3a* and *Axin2* overlap significantly (Fig. S4). Expression of W*nt3a* in *Brachyury* homozygous mutant embryos at this stage resembles that in heterozygous and wild type individuals, as has been reported previously [40], but by E8.5, when *Wnt3a* expression is restricted to the primitive streak, its expression is significantly down regulated in *Brachyury* mutant embryos (Fig. 4C,D).

Like *Wnt3a*, *Axin2* is expressed in *Brachyury* mutant embryos at 7.5 dpc, but this expression is more variable than that of *Wnt3a*, and is sometimes reduced or even absent (data not shown). By E8.5, when *Axin2* is expressed in the headfold, tailbud and primitive streak of wild type embryos, its expression in the posterior region of *Brachyury* mutant embryos is very weak or absent (Fig. 5C,D). Together, these data indicate that Brachyury is required for the proper expression of *Wnt3a* and *Axin2*, which encode key components of the WNT signalling pathway (see Discussion).

### *Fgf8* as a target of Brachyury

**Figure 6:**
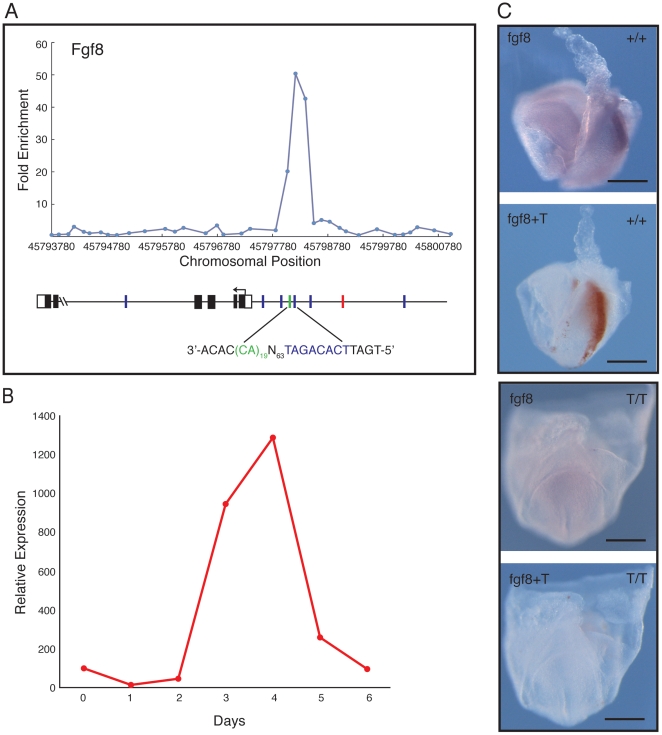
*Fgf8* as a target of Brachyury. (A) Location analysis of *Fgf8*. For details of methods see legend to Fig. 4. (B) Quantitative RT-PCR expression profile for *Fgf8* during ES cell differentiation, expressed relative to *beta actin*. (C) Expression of *Fgf8* studied by in situ hybridisation. The images show a phenotypically wild type (+/+ or *+*/*T*) embryo (top pair) and a mutant *T*/*T* (bottom pair) embryo derived from crosses of *Brachyury* heterozygous mutant mice. The wild type embryo is orientated with anterior to the left and posterior to the right; the mutant is viewed from the posterior. *Fgf8* expression is detected with NBT/BCIP (purple) and *Brachyury* with INT/BCIP (orange brown). In the wild type embryo *Fgf8* is expressed in the primitive streak and paraxial mesoderm; such expression is absent or greatly reduced in the mutant. Scale bars indicate 200 µm.

A strong Brachyury binding peak was also detected 5′ of the transcription start site of *Fgf8* (Fig. 6A), with a variant Brachyury site (5′-TCACAGAT-3′; underlined bases differ from consensus) positioned 63 nucleotides from an (AC)_19_ repeat. The temporal expression profile of *Fgf8* resembles that of *Brachyury* during embryoid body differentiation (Fig. 6B), and the gene is co-expressed with *Brachyury* in the primitive streak of embryos at E7.5 and E8.0–E8.25 (Fig. S5; Fig. 6C). Expression of *Fgf8* in mutant *Brachyury* embryos at E8.0 is greatly reduced (Fig. 6C), indicating that Brachyury is required for expression of this gene as it is for *Wnt3a* and *Axin2*.

### *AXIN2, JUP, FGF8 and WNT3A are* conserved targets of BRACHYURY in the human

Because some of the Brachyury targets we discovered in the mouse were not previously identified in the frog or the zebrafish [16], we decided to investigate whether these are conserved in other species, in an attempt to further validate our results. For this purpose we decided to look for BRACHYURY binding in the human genome.

We have recently optimised culture conditions that cause human embryonic stem cells to differentiate into mesoderm-like cells [41]. These cell populations express BRACHYURY at high levels and, importantly, they also up regulate other mesoderm markers, including many of the Brachyury targets we have identified in the mouse, namely, *DKK1*, *HOXA13*, *ID4*, *JUP*, *KRT8*, *MEIS1*, *MSGN1*, *SNAI2*, and *WNT3A*.

**Figure 7:**
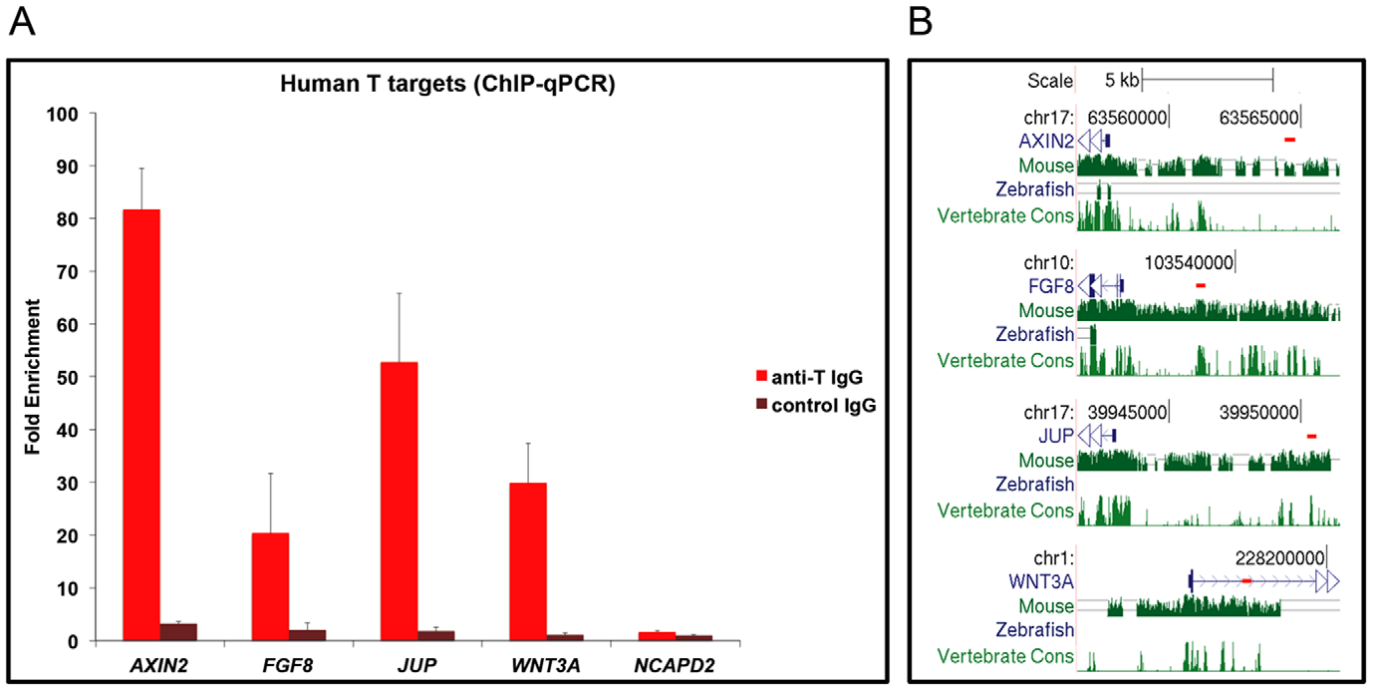
Conservation of BRACHYURY binding in the human genome. (A) ChIP-qPCR performed on samples from differentiated hECSs using a specific anti-BRACHYURY IgG and a non-specific control IgG. Graph shows enrichment for regulatory regions of Brachyury targets (*AXIN2*, *FGF8*, *JUP*, *WNT3A*) and a negative control region (*NCAPD2*). Results are expressed relative to input chromatin divided by the enrichment for the non-specific control antibody. (B) BRACHYURY binding in the human genome. The short red lines below the chromosomal coordinates (hg19) depict the position of the PCR amplicons relative to the beginning of the human genes (blue). The three bottom tracks show the genome sequence conservation between human and mouse, zebrafish and vertebrate genomes (Genome Browser, http://genome.ucsc.edu/).

We therefore made use of this newly developed *in vitro* differentiation system to ask if BRACHYURY binds the homologous human regulatory regions of some key mouse targets: *AXIN2*, *FGF8*, *JUP* and *WNT3A*. As in the mouse, these regions contain imperfect T-binding motifs (data not shown). Our experiments involving ChIP-qPCR with hESC-derived mesoderm cells indeed detected a strong enrichment for these sequences, thus indicating that BRACHYURY binds to the same genomic regions in the human (Fig. 7A).

Interestingly, these promoter sequences seem to be conserved between the mouse and human genomes, but not in the zebrafish or in other vertebrates (Fig. 7B) suggesting that these targets might be unique to mammals.

## Discussion

We have identified genomic targets of Brachyury in differentiating mouse ES cells, demonstrating that embryoid bodies provide sufficient material for chromatin immunoprecipitation experiments and that they represent an effective model of early mouse development. Although they do not undergo proper morphogenesis, they do generate pattern, as illustrated by the formation of beating cardiomyocytes [42]. The embryoid bodies produced in our experiments form cardiomyocytes after eight to ten days in culture, close to the time at which the heart tube forms during normal development. As discussed below, at least three Brachyury targets (*Wnt3a*, *Axin2* and *Fgf8*) are expressed in the early mouse embryo during formation of the primitive streak, and their proper expression during development requires *Brachyury* function. We also note that several targets are expressed in primordial germ cells, perhaps the first differentiated population to emerge during early gastrulation [43], [44], [45]. These cells express *Brachyury* until E12.5, when the gene is down regulated in a non-migrating population [46], [47]. Although *Brachyury* may not be involved directly in the specification of the germ cells [48], [49] it may regulate their migration and their potency.

### Classification of Brachyury targets and comparison with zebrafish and frog

Our work has identified 396 potential targets of Brachyury, and gene ontology analysis indicates that many of these encode sequence-specific DNA binding proteins and proteins involved in cell adhesion and embryonic morphogenesis. Analysis of the former category will help in the elucidation of the genetic regulatory network that underlies mesoderm formation (see below). The latter category includes cell junction proteins and glycosyltransferases [31], consistent with the finding that the extracellular matrix of homozygous mutant *Brachyury* mouse embryos is poorly developed [32] and that cells have fewer cytoplasmic processes, both of which may contribute to the failure of mutant cells to move out of the primitive streak and to the failure of elongation of the antero-posterior axis [30]. Amongst the other genes regulated by Brachyury are those encoding cytokines and components of signal transduction pathways, and in most of these respects our results are reminiscent of those obtained in similar experiments using the zebrafish embryo [16].

It is significant that embryoid bodies resemble developing embryos in this way, and it is also important to note the overlap between the Brachyury targets identified in this study and the Ntl targets identified in the zebrafish [16]. Both studies identified transcriptional regulators as being enriched, including members of the homeobox, winged helix, paired box, zinc finger and odd-paired families. There are also similarities in the functions of genes regulated by the two orthologues. These functions include gastrulation (where the zebrafish study identified *wnt11, snail1a* and *blf* and this analysis *Wnt3a*, *Snail2* and genes such as *Gdf5*, *Etv1*, *Krt5*, *Krt8*, *Lmx1b*, *Syk*, and *Gnaq*); muscle specification (where both studies identified *Msgn1* and *Pax3*); posterior identity (where the zebrafish study identified *fgfr4, fgfr28, vent, vox*, and *notch3* and this analysis *Fgf8* [50]) and left-right patterning (zebrafish genes include *cx43.4* [51] and our mouse targets *Rttn* [52], *Fgf8* [53], and cytoplasmic dyneins *Dync1li1*, *Dync2li1* [54] and *Dpcd* [55]).

The fact that there are some differences between the mouse and zebrafish targets may derive from the presence of an additional *Brachyury* gene in the zebrafish genome [8] or from the ‘sharing’ of gene function between different T box family members. For example, the *Bix* genes were identified as targets of Brachyury and VegT in *Xenopus* [13], [14], but their mouse ortholog *Mixl1* seems to be regulated mainly by Eomesodermin [56], [57]. Furthermore, as illustrated in Fig. 7B, some regulatory sequences of mammalian genes (*Axin2*, *Fgf8*, *Jup* and *Wnt3a*) share little homology with those of their zebrafish orthologues. It is possible that Brachyury binds different locations in different genomes, which has been noted for other transcription factors [58], despite target conservation. It is also likely that Brachyury binds not only to promoters near the gene transcription start site but also to distant enhancers [59], which is indeed the case in the human genome (T. Faial *et al*., in preparation). We note that both the mouse and zebrafish arrays were based on promoter regions, so that enhancer binding is not available in these datasets, perhaps explaining why some targets seem to be unique to each species.

### Canonical and non-canonical T-box binding sites

Our previous work searching for targets of zebrafish Ntl showed that the canonical T-box site TCACACCT was enriched in the vicinity of Ntl target genes [16]. A significant enrichment of this motif was not observed in the present experiments for the majority of targets. Rather, we identified a novel (AC)_ n_ repeat sequence that recognised, albeit weakly, the Brachyury T domain in electrophoretic mobility shift experiments. We do not yet fully understand the significance of this observation. Mouse Brachyury binds to an imperfect T-box site palindrome in the *Nanog* promoter [60], but no other Brachyury target has been characterised in any detail in this species. It is possible that mouse Brachyury resembles *Drosophila* Brachyenteron, where modular variations on the T-box consensus binding sequence determine the degree of transcriptional activation [61]. A similar system controls notochord formation in *Ciona*, with regulatory motifs comprising Ci-Brachyury and Ci-foxA binding sites [62].

Moreover, many transcription factors bind directly to DNA in distal enhancer elements [59], and are then linked to the promoter region by chromatin looping, allowing interaction with other proteins involved in transcription regulation [59]. It is likely that in some mouse targets, canonical Brachyury binding motifs are not present in the promoter region but rather in upstream or downstream regulatory regions. Our results show that this does occur in the human genome (T. Faial *et al*., in preparation).

It is also possible that the AC repeats cause the transient formation of left handed DNA helices and bends, changing the chromatin architecture and encouraging transcription factor binding [63]. Brachyury may be an example of a protein with a secondary recognition motif [64] and that the presence of both an AC repeat and a TCACACCT sequence allows stable binding that cannot be competed by an excess of just the TCACACCT sequence (Fig. S3B). Repetitive sequences may also function as pre-sites; that is, as regions of DNA that are predisposed to evolve into new regulatory sequences [65].

### Brachyury modulation of Wnt and Fgf signalling

Several components of the Wnt signal transduction pathway were identified as Ntl targets in the zebrafish, and we find that the same is true for Brachyury in the mouse. In an effort to determine whether Brachyury regulates expression of these potential targets during normal mouse development we asked whether *Wnt3a* and *Axin2* are expressed normally in *Brachyury* homozygous mutant embryos, and found that although both genes are expressed at E7.5 (albeit rather variably in the case of *Axin2*), neither is expressed at E8.5 in mesodermal derivatives (Figs. 4, 5). This suggests that Brachyury is not required for the initial activation of *Wnt3a* or *Axin2*, but is needed for maintenance of their expression. Together with the observation that Wnt3a maintains *Brachyury* expression in the early mouse embryo via TCF/Lef signalling [20], [21], and that *Axin2* is down regulated in *Wnt3a* mutants [66], our data indicate that Brachyury and Wnt signalling cooperate to create a regulatory network that specifies the formation of posterior mesoderm in the mouse embryo.

Part of this network may involve Fgf signalling. Brachyury and Fgf signalling form part of an autoregulatory loop in *Xenopus* and zebrafish embryos [67], [68], [69], [70], and we note that *Fgf8* is a target of Brachyury in embryoid bodies, and that its expression is down regulated in *Brachyury* mutant embryos (Fig. 6).

Finally, our work reveals that the promoter regions of *AXIN2*, *FGF8* and *WNT3A* are also bound by BRACHYURY (Fig. 7A) in human ES cells as they differentiate into mesoderm-like cells [41]. These results further substantiate the identity of these genes as *bona fide* Brachyury targets and suggest that the regulation of these key signalling components is conserved during human development.

### Making a genetic regulatory network for mesoderm

Attempts to understand the Brachyury genetic regulatory network are important not only because Brachyury is required for proper formation of mesoderm in the vertebrate embryo, but because it is sufficient for the formation of some mesodermal cell types, at least in *Xenopus* [71]. The identification of new Brachyury targets will enable the integration of Brachyury with other components of genetic regulatory networks that include it, such as the Ets family member Elk-1 and the caudal homologue, Cdx2 [72] and to ask to what extent such networks have been conserved during evolution.

## Materials and Methods

### Ethics statement

Animal procedures were performed under a UK Home Office project license within the conditions of the Animals (Scientific Procedures) Act 1986.

### Mouse ESC culture

Embryonic stem (ES) cell culture was as described [73] except that mitotically inactivated primary mouse embryonic fibroblasts (MEFs) were used as feeders. Culture dishes were coated with 0.1% gelatin (Sigma-Aldrich). MEFs and ES cells were maintained in DMEM (Sigma-Aldrich) supplemented with 0.1 mM β-mercaptoethanol, non-essential amino acids (Gibco Invitrogen), 2 mM glutamate (Gibco Invitrogen), and batch-tested 10% (MEFs) or 15% (ES cells) foetal bovine serum (FBS) (Gibco Invitrogen). ES cell medium was also supplemented with Leukaemia Inhibitory Factor (LIF) (ESGRO®, Millipore) at 10^3^ units/ml [74]. Early passage R1 mouse ES cells [75] were passaged every 2 days and medium was changed daily to prevent differentiation.

ES cells were differentiated in spinner flasks to produce large numbers of embryoid bodies (EBs) undergoing synchronized differentiation [76], [77]. The Cellspin culture system (Integra Biosciences) was set at 25 rpm and spin angle 720° so as to avoid aggregation of EBs. Spinner medium was prepared as above, but with LIF omitted and FBS increased to 20%. On day 0, adherent log phase ES cell colonies were dissociated and resuspended in 10 ml spinner medium. Feeder cells were depleted by differential sedimentation at 37°C for 20 min.

Medium (45 ml) was pre-equilibrated in 100 ml silicon-coated (Sigmacote, Sigma-Aldrich) spinner flasks (Integra Biosciences). ES cells were recovered from the gelatin-coated differential sedimentation plates and centrifuged at 800 g for 5 min. Cells were fully dissociated to ensure that cultures were initiated from single cells, and each spinner flask was inoculated with 10^7^ cells in 5 ml medium. After 24 h (day 1 of differentiation) a further 50 ml spinner medium was added to each flask. Each day thereafter EBs were allowed to sink and 50 ml medium was aspirated and replaced with 50 ml fresh pre-warmed spinner medium.

### Human ESC culture

Human ESCs (H9 [WiCell, Madison, WI]) were maintained and differentiated as previously described [41]. Briefly, hESCs were induced to express BRACHYURY by culturing them in a chemically defined medium (CDM) supplemented with FGF2 (20 ng/ml), LY294002 (10 µM) and BMP4 (10 ng/ml) (termed FLyB medium). Cells were collected for ChIP after 36 h of culture in FLyB medium, when BRACHYURY expression peaked.

### Quantitative RT-PCR

Gene expression was analysed by real-time RT-PCR. RNA was isolated from differentiated EBs using Tri-Reagent LS (Sigma-Aldrich), digested with DNA-free DNAse I (Ambion), and checked for integrity using an Agilent Technologies 2100 Bioanalyser. cDNA was generated from l µg RNA using Superscript III Reverse Transcriptase (Invitrogen, Life Technologies), and this was followed by real-time PCR using the LightCycler 480 SYBR Green I master kit (Roche). Mouse *beta actin* primers were used as an endogenous control to express relative expression levels (Table S6).

### Antibodies

Several anti-Brachyury antibodies were tested for use in this work. Of these, the goat polyclonal C19 antibody (SC-17745, Santa Cruz Biotechnology), raised against a C-terminal sequence of human Brachyury, performed best in chromatin immunoprecipitation. This antibody, raised against a divergent region of Brachyury that does not include the T box, has been well characterised in previous studies [78], [79], [80]. It gave the expected pattern of staining in early mouse embryos (Fig. S6A,B) and recognised Brachyury protein (of the correct size) in immunoprecipitation experiments followed by western blots (Fig. S6C). Such experiments failed to detect Brachyury in ES cells in which Brachyury expression was inhibited by use of ShRNA constructs (Fig. S6D,E).

### Whole-mount *in situ* hybridization

Wild type mouse embryos were collected from MF1 or 129 strains, and *Brachyury* mutant embryos from BTBR T^+^tT/J×BTBR T^+^tT/J heterozygote crosses [39]. Embryos were fixed overnight in 4% paraformaldehyde in phosphate-buffered saline (PBS), after which they were dehydrated and stored in 100% methanol at −20°C. The mouse Brachyury coding sequence was subcloned into pCS2+ and used to generate a probe. An *Axin2* probe was generated from IMAGE clone 1361800 (Geneservice), a *Wnt3a* probe from IMAGE clone pENTR223.1 100015989 after subcloning into pCS2+ (Table S7), and an *Fgf8* probe from IMAGE clone 6513131 (Geneservice) in pCMV-SPORT 6.1. Digoxigenin labelled or fluorescein labelled antisense RNA probes were generated using T7 RNA polymerase from linearised templates and whole mount in situ hybridisation was performed as described [81]. Alkaline phosphatase was detected using (i) BM purple; (ii) 2-[4-iodophenyl]-3-[4-nitrophenyl]-5- phenyltetrazolium chloride (250 µg/ml) plus magenta phosphate (250 µg/ml) (INT/Mag); or (iii) nitro blue tetrazolium (175 µg/ml) plus 5-bromo-4-chloro-3-indolyl phosphate (337.5 µg/ml) (NBT/BCIP) (Roche). These gave dark blue, orange brown or purple staining respectively. A final concentration of 5% polyvinyl alcohol (Sigma-Aldrich) was used in the staining reaction.

### Whole-mount immunohistochemistry

Embryos were fixed as described above and rehydrated to PBS for staining. Free aldehyde groups were blocked using 1 M glycine, embryos were washed in PBS/0.1% Tween 20 (PBST), and endogenous peroxidases were blocked using 3% hydrogen peroxide in PBS. Embryos were incubated overnight at 4°C in 1∶400 C19 antibody in PBST supplemented with 0.2% bovine serum albumin (BSA) and 10% heat inactivated FBS. They were then washed, incubated with 1∶400 rabbit anti-goat biotinylated IgG (E0466, Dako), and stained using Vectastain Elite ABC substrate (Vector laboratories) with Sigma Fast Nickel Enhanced DAB chromagen (Sigma).

### In vitro translation and western blotting

*Brachyury* mRNA was synthesized using the pCS2+ construct described above and the Ambion mMessage mMachine (Applied Biosystems/Ambion). mRNA was translated in a rabbit reticulocyte lysate (Promega). In vitro translation products and embryoid body extracts were subjected to polyacrylamide gel electrophoresis (PAGE) and western blots were performed using CAPS transfer buffer (10 mM CAPS pH 11, 10% Methanol). Membranes were blocked with 5% milk powder in PBST overnight, and antibodies were diluted in the same solution. Washes were in PBST. Primary antibodies were R&D Systems anti-T and SantaCruz anti-T (see above). Both were used at a dilution of 1∶250. Secondary antibodies were HRP-linked SantaCruz D anti-goat IgG (1∶20,000) and HRP-linked Amersham NA934V anti-rabbit IgG (1∶100,000). All antibody incubations were 1 hour at room temperature. Both endogenous and *in vitro* translated T proteins were immunoprecipitated for western blotting using the Santacruz Exactacruz D anti-goat system (SC-45041, Santa Cruz) to avoid detection of heavy and light chains of the IP antibody. Detection used the Pierce Supersignal West Dura Extended Duration Substrate (Thermo Scientific).

### Chromatin immunoprecipitation (mouse ESCs)

Chromatin immunoprecipitation/location analysis was based on the Agilent Mammalian ChIP-chip Protocol, incorporating the Whole Genome Amplification GenomePlex Kit (Sigma) [82]. Intact EBs (1.5×10^9^ cells) were fixed in 1 M formaldehyde for 20 min when *Brachyury* expression was at its highest level (usually after 4 days of differentiation). This was followed by quenching and isolation of nuclei. Our protocol differs from a previously-published procedure [83] in that EBs are not disrupted before fixation. Nuclei were sonicated using a Misonix 3000 ultrasonicator to create fragments of 500 bp, and these were immunoprecipitated using polyclonal goat anti-Brachyury C-19 (Santa Cruz Biotechnology) or normal goat IgG (Santa Cruz Biotechnology) as an isotype control. Following washing and elution steps, cross-links were reversed overnight at 65°C. Samples were analysed by promoter-specific primers or amplified by GenomePlex whole genome amplification for microarray studies.

### Chromatin immunoprecipitation (human ESCs)

ChIP was performed as previously described [83] with some modifications. Briefly, H9 hESCs (one confluent 10 cm dish) were collected after 36 hr of culture in FLyB medium [41], when BRACHYURY expression peaked. Cells were fixed as described [83], the nuclei were isolated and sonicated using a Misonix 4000 to obtain DNA fragments of around 1000 bp. Samples were incubated at 4°C overnight using 10 µg of an anti-BRACHYURY goat IgG (R&D systems) and with10 µg of a non-specific goat IgG as a control. The chromatin was immunoprecipitated by adding 100 µl of Protein G Dynabeads (Invitrogen), then incubating at 4°C 1 h, and collecting the beads using a magnetic rack. After washing the beads, the chromatin was eluted and the crosslinking was reversed at 65°C overnight. Samples were then treated with RNAse and Proteinase K and the DNA was extracted by phenol/chloroform, ethanol-precipitated and finally eluted in nuclease-free water. This experiment was repeated three times with similar results.

Verification of target enrichment was performed on a selection of targets using genomic quantitative PCR. DNA fragments were amplified using Fast SYBR® Green Master Mix (Applied Biosystems) according to manufacturers instructions on a 7500 Fast Real-Time PCR System (Applied Biosystems). Promoter specific primers (Table S8) were designed to amplify the homologous regions of mouse T-binding sites (AXIN2, FGF8, JUP and WNT3A). *NCAPD2* was used as a negative control gene.

### Microarray hybridization, analysis and verification of binding targets

Agilent Technologies mouse promoter 244K (G4490A) 60-mer oligonucleotide arrays (“chips”) covering 17,000 mouse genes and extending 5.5 kb upstream and 2.5 kb downstream of transcriptional start sites were hybridized with 5 µg amplified chromatin per sample. Arrays were annotated to NBI35.1 of the mouse genome. Immunoprecipitated (or isotype control) and total input samples were labelled with Cy5 or Cy3 respectively. Hybridizations were performed and analysed in triplicate using independently differentiated cultures. The isotype control experiment was performed once to confirm no significant enrichment over input chromatin (data not shown).

Microarrays were scanned using an Agilent scanner to a resolution of 5 µm. Data were extracted using Agilent G2567AA Feature Extraction Software (v.9.1). The significance of binding events was determined using Agilent Chip Analytics 1.3 software. Initial analysis was done using Chip Analytics defaults settings and then further filtered using the parameters P(*x*) <0.01 and P(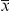) <0.005. The confidence of binding calls is represented as a P-value: P(*x*) defines the value for each probe, and P(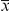) uses the intensities of neighbouring probes to assess peak shape, in an effort to eliminate false positives. Original raw data files can be accessed from GEO Gene Expression Omnibus http://www.ncbi.nlm.nih.gov/geo (accession GSM417692/GSM417704 for design 1 and 2 Brachyury data; GSM417714/GSM417756 for design 1 and 2 isotype control data).

Verification of enrichment was performed on a selection of targets using promoter specific genomic quantitative PCR. Promoter specific primers (Table S8) were designed so as to span bound peaks using mouse build mm8 promoter sequence retrieved from the UCSC genome browser (http://genome.ucsc.edu/). Negative control genes from the list not called as bound were included. Results were expressed relative to input chromatin divided by relative enrichment for the isotype control antibody.

### Bioinformatic analyses and Motif Finding

The GOToolBox (http://burgundy.cmmt.ubc.ca/GOToolBox/) [84] was used to access Gene Ontology (GO) resources and to search for any functional bias in our dataset. The Benjamini and Hochberg multiple testing correction was applied to assess the significance of enrichment ratios. Target probes and surrounding promoter sequences were scanned for the published consensus *in vitro* T-box binding motif TCACACCT [17], [18] using NestedMICA http://www.sanger.ac.uk/Software/analysis/nmica/index.shtml [85]. We also used Regulatory Sequence Analysis Tools (RSAT) http://rsat.ulb.ac.be/rsat/ [86], to scan each target gene over a region −5 kb to +1 kb relative to its ATG for over-represented cis-regulatory modules, applying background models and taking promoter sequences from 400 random mouse promoters as the control set. Sequences representing enriched motifs were then stacked into positional weight matrices and converted to sequence logos using WebLogo (http://weblogo.berkeley.edu/logo.cgi) [87].

### Electrophoretic mobility shift assays

The T domain of mouse *Brachyury* was amplified by PCR (primer sequences in Table S7) and inserted in-frame into the glutathione-S-transferase (GST) fusion vector pGEX-6P-1. The fusion protein was expressed in *E. coli* by isopropyl-β-D-thiogalactoside induction and purified at 4°C using GSTrap FF columns and Pre-Scission Protease (GE Healthcare), leaving only a glycine and a proline residue attached to the protein. This was concentrated using Amicon Ultra 4 columns (Millipore), and the identity of the resulting protein was confirmed by SDS PAGE and mass spectrometry.

Double stranded oligomers containing (i) the core Brachyury consensus binding sequence TCACACCT, (ii) a simple AC repeat, or (iii) the core Brachyury sequence together with the AC repeat, and mutated versions of each, had identical BglII/BamH1 5′ overhangs (Table S7). These were PAGE purified, annealed, and end-labelled with [α-^32^P] dCTP using Klenow fragment. Unincorporated nucleotides were removed using Sephadex G-50 columns (GE Healthcare). Binding reactions were incubated on ice for 40 minutes in 1× EMSA binding buffer (25 mM HEPES pH8.0, 100 mM KCl, 1 mM DTT, 0.1% NP-40, 5% glycerol, 10 mM EDTA), 0.5% milk powder and 50 ng/µl dI/dC using 30,000 cpm/µl and 8–10 fmol labelled oligomer. Competition reactions using 4 pmol cold oligomers were pre-incubated for 10 min on ice. In supershift experiments goat polyclonal anti-Brachyury N19 antibody (SC-17743, Santa Cruz) was added after binding and then incubated a further 20 min on ice.

## Supporting Information

Figure S1 **Validation of targets.** Box plot showing genomic quantitative PCR of bound promoter regions for targets *Axin2*, *Foxe1*, *Mapre2*, *Nkx2.6*, *Pax3*, *Rttn*, *Van Gogh* and the published target *Nanog*, and unbound or negative promoter regions *Nanog 3′*, *1700010C24Rik* and *beta actin*. Boxes represent the interquartile range, the upper edge being the 75^th^ percentile and lower edge the 25^th^ percentile. The whiskers show the minimum and maximum values. Values above the line are enriched in chromatin immunoprecipitations. Data were obtained from five independent chromatin immunoprecipitations. Probes recognising *Nanog* were not present on Agilent 244K promoter arrays.
(TIF)

Figure S2 **Functional analysis of target genes.** Bar charts show Gene Ontology (GO) annotations for (A) biological process; (B) cellular component; and (C) molecular function using the GOToolBox. Horizontal bars represent enrichment ratio (observed frequency/expected frequency) and vertical axis gives the GO term followed by the GO identification number in brackets and hierarchy level. Colour bars indicate statistical significance. GO terms related to the function of Brachyury are highlighted in red boxes.
(TIF)

Figure S3 **Interaction of the mouse Brachyury T domain with DNA.** (A) Sequences surrounding bound probes are enriched for an (AC)_n_ repeat relative to their genomic neighbours. The motif was generated using the NestedMICA position weight matrix. This may represent a secondary Brachyury recognition motif. (B) Electrophoretic mobility shift assays. Panel a: Binding reactions using ^32^P-labelled TCACACCT. Lane 1, no protein; lane 2, control protein derived from empty vector; lanes 3–6, mouse T domain protein: lane 4 includes excess unlabelled probe; lane 5 includes excess unlabelled mutated probe; lane 6 is a ‘supershift’ using anti-T N-19 (SC-17743, Santa Cruz). Notice that the Brachyury T domain binds the T site oligonucleotide and that binding is competed by cold wild-type oligonucleotide but not by a mutated oligonucleotide. Panel b: Lanes 7–9 include ^32^P-labelled TCACACCT; lane 10 uses the indicated mutated version of this oligonucleotide. Note that the Brachyury T domain does not bind the mutated oligonucleotide. Panel c: Binding reaction using a ^32^P-labelled AC repeat oligonucleotide. Lanes 11–13 as panel a; lane 14 includes excess unlabelled probe; lane 15 includes excess of an unlabelled mutated probe; lane 16 is a ‘supershift’. Notice that the Brachyury T domain binds the AC repeat oligonucleotide weakly but that binding does not seem to be competed by cold wild-type oligonucleotide. The complex however is ‘supershifted’ using the Brachyury antibody. Panel d: Binding reactions using a ^32^P labelled motif that includes both the T site TCACACCT and an AC repeat. Lanes 17–20 show that Brachyury binds this oligonucleotide, and that binding is competed by cold wild-type oligonucleotide. Lanes 21 and 22 show that binding is not competed significantly by unlabelled oligonucleotides in which either motif is mutated. Lane 23 shows a ‘supershift’. Experiments in (a–d) were performed under identical conditions and exposed for the same times.
(TIF)

Figure S4 **The expression domains of** ***Brachyury*** **,** ***Wnt3a*** **and** ***Axin2*** **overlap in E7.75 mouse embryos.** (A) Expression of *Axin2* analysed using a fluorescein labelled antisense probe detected with NBT/BCIP (purple). (B) The embryo in (A) analysed using a digoxigenin labelled antisense *Brachyury* probe detected with INT/Mg phosphate (brown). (C) Expression of *Wnt3a* analysed using a fluorescein labelled antisense probe detected with NBT/BCIP (purple). (D) The embryo in (C) analysed using a digoxigenin labelled antisense *Brachyury* probe detected with INT/Mg phosphate (brown). All embryos orientated as in (A). Scale bars are 200 µm.
(TIF)

Figure S5 **The expression domains of** ***Brachyury*** **and Fgf8 overlap in the primitive streak of E7.75 mouse embryos.** (A). Expression of *Fgf8* analysed using a fluorescein labelled antisense probe detected with NBT/BCIP (purple). (B). The embryo in (A) analysed using a digoxigenin labelled antisense *Brachyury* probe detected with INT/Mg phosphate (brown). Black bars are 200 µm.
(TIF)

Figure S6 **Verification of anti Brachyury antibody.** (A) Immunohistochemistry of E9.5 embryo using Santa Cruz anti-human T C19 with nickel enhanced DAB substrate. Staining is present in the notochord (arrowhead), pre-somitic mesoderm (arrow) and tailbud. Staining was absent in controls in which primary or secondary antibodies were omitted. (B) Expression of *Brachyury* RNA in an E9.5 embryo studied by in situ hybridisation. Note similarity to (A). Bars in (A) and (B) represent 250 µm. (C) Western blot testing antibody specificity. Size markers are shown to the left. Lane 1: Mouse Brachyury reticulocyte lysate translation product; lane 2: unprogrammed reticulocyte lysate translation product; lane 3: Immunoprecipitated material derived from Brachyury reticulocyte lysate translation product; lane 4: Supernatant of immunoprecipitated material in lane 3; lane 5: Immunoprecipitated material derived from day 4 embryoid bodies; lane 6: Supernatant of immunoprecipitated material in lane 5; lane 7: Immunoprecipitated material derived from day 4 embryoid bodies, having omitted first antibody; lane 8: Supernatant of immunoprecipitated material in lane 7. All immunoprecipitations used Santa Cruz anti-T C19. Western blots used R&D Systems anti-T as a primary antibody and SantaCruz D anti-goat IgG HRP linked secondary antibody. (D) Strategy to create ES cell clones lacking Brachyury. Clones were created using 65 bp ShRNA duplexes targeting the first exon of *Brachyury* (*T*). Sequences were inserted into the XhoI/HindIII site of the pSingle ShRNA vector (Clontech) which includes a tetracyclin-controlled transcriptional repressor that in turn regulates the expression of the ShRNA sequence. Selection of stable lines is achieved by culture in G418 and induction of ShRNA expression occurs through addition of 1 µg/ml doxycycline. (E) Western blot analysis of day 5 embryoid body extracts from clones containing ShRNA constructs targeted to *Brachyury* exon 1 (T1) or a scrambled version of this sequence (Ts), either treated with doxycycline (+) or left untreated (−). Samples were immunoprecipitated as in (C). Note loss of Brachyury band in lane 3.
(TIF)

Table S1 **Full gene list.**
(DOC)

Table S2 **Targets involved in key signalling pathways.**
(DOC)

Table S3 **Genes associated with germ cell development.**
(DOC)

Table S4 **Targets identified as transcription factors.**
(DOC)

Table S5 **Conservation of AC repeats.**
(DOC)

Table S6 **Quantitative PCR primers.**
(DOC)

Table S7 **In situ hybridisation, T box, and electrophoretic mobility shift assay primers.**
(DOC)

Table S8 **Genomic quantitative PCR primers.**
(DOC)
