# Supplementary figures and images for: Genomic Targets of Brachyury (T) in Differentiating Mouse Embryonic Stem Cells
Source: PLoS One. 2012 Mar 30;7(3):e33346. doi: 10.1371/journal.pone.0033346 (PMC3316570; doi:10.1371/journal.pone.0033346)

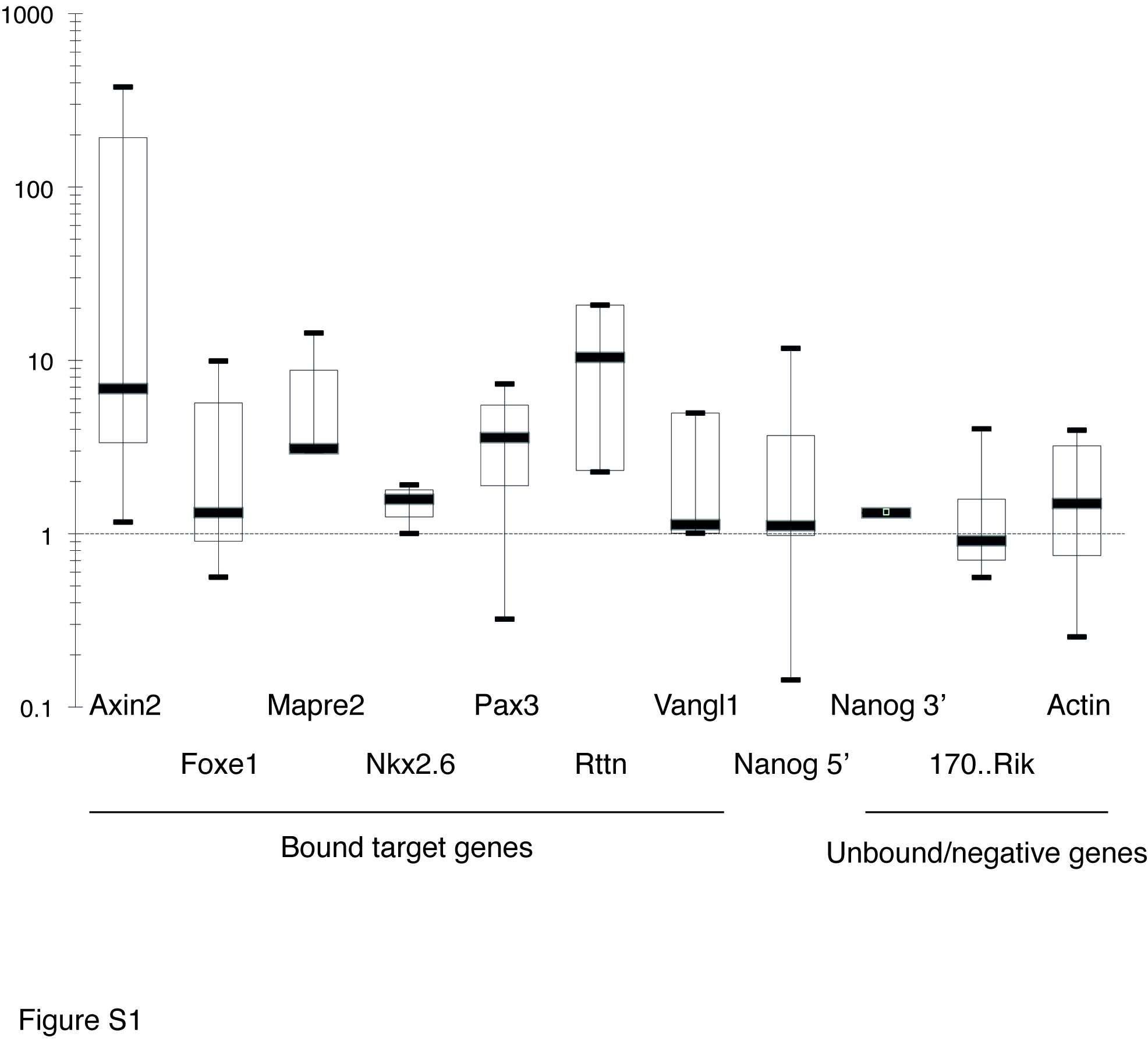

Supplement: Figure S1 — Validation of targets. Box plot showing genomic quantitative PCR of bound promoter regions for targets Axin2, Foxe1, Mapre2, Nkx2.6, Pax3, Rttn, Van Gogh and the published target Nanog, and unbound or negative promoter regions Nanog 3′, 1700010C24Rik and beta actin. Boxes represent the interquartile range, the upper edge being the 75th percentile and lower edge the 25th percentile. The whiskers show the minimum and maximum values. Values above the line are enriched in chromatin immunoprecipitations. Data were obtained from five independent chromatin immunoprecipitations. Probes recognising Nanog were not present on Agilent 244K promoter arrays. (TIF) [file pone.0033346.s001.tif]

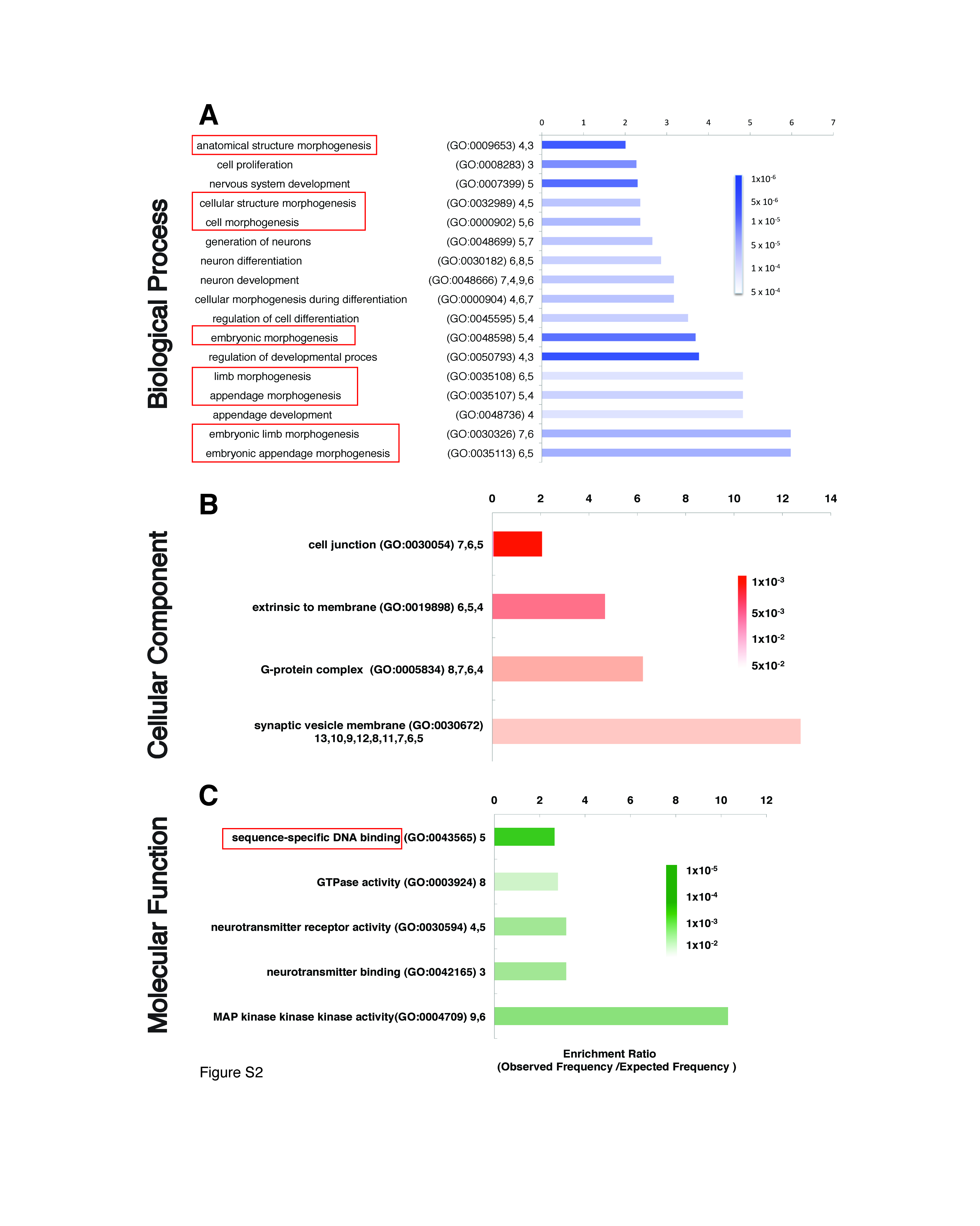

Supplement: Figure S2 — Functional analysis of target genes. Bar charts show Gene Ontology (GO) annotations for (A) biological process; (B) cellular component; and (C) molecular function using the GOToolBox. Horizontal bars represent enrichment ratio (observed frequency/expected frequency) and vertical axis gives the GO term followed by the GO identification number in brackets and hierarchy level. Colour bars indicate statistical significance. GO terms related to the function of Brachyury are highlighted in red boxes. (TIF) [file pone.0033346.s002.tif]

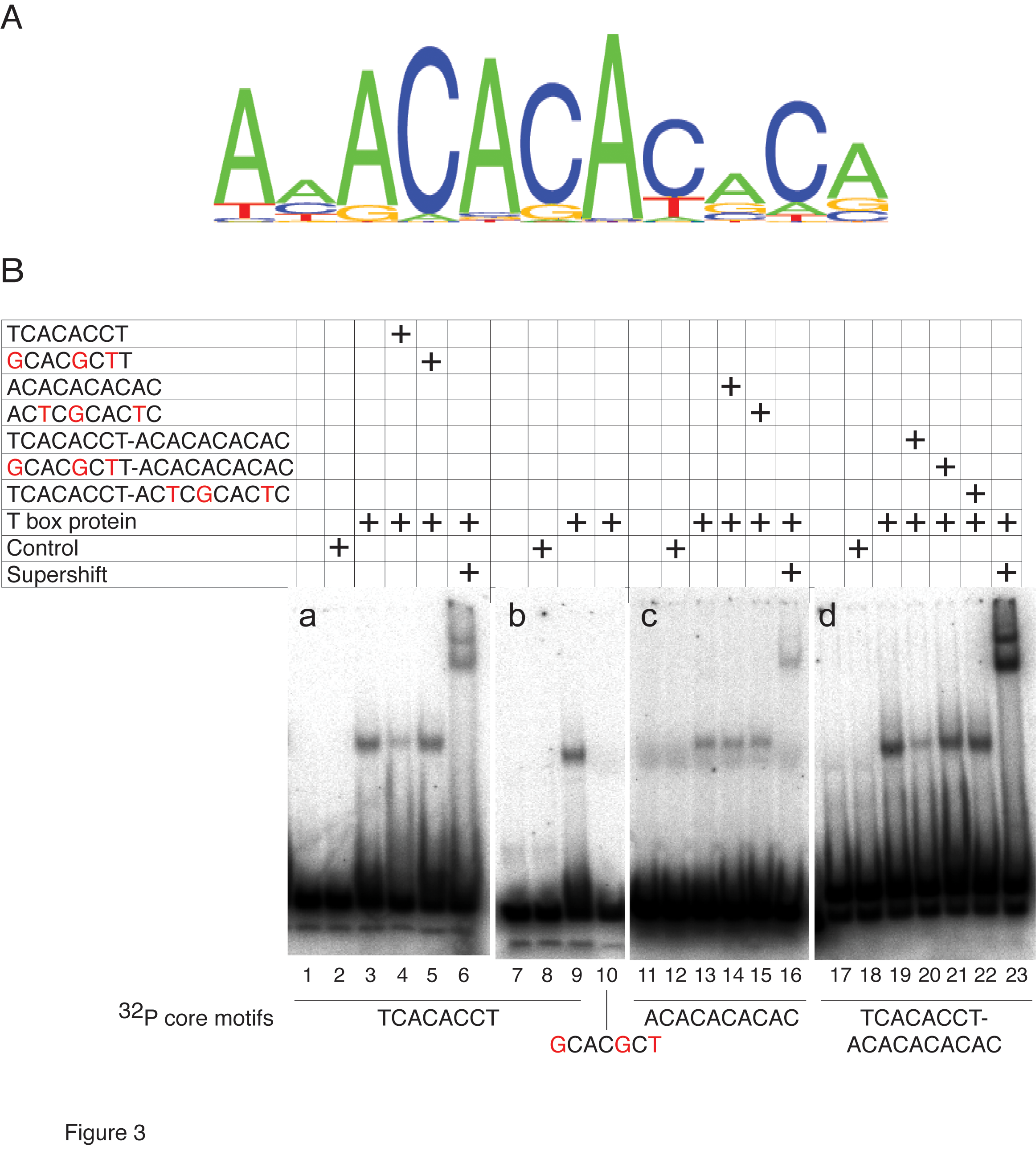

Supplement: Figure S3 — Interaction of the mouse Brachyury T domain with DNA. (A) Sequences surrounding bound probes are enriched for an (AC)n repeat relative to their genomic neighbours. The motif was generated using the NestedMICA position weight matrix. This may represent a secondary Brachyury recognition motif. (B) Electrophoretic mobility shift assays. Panel a: Binding reactions using 32P-labelled TCACACCT. Lane 1, no protein; lane 2, control protein derived from empty vector; lanes 3–6, mouse T domain protein: lane 4 includes excess unlabelled probe; lane 5 includes excess unlabelled mutated probe; lane 6 is a ‘supershift’ using anti-T N-19 (SC-17743, Santa Cruz). Notice that the Brachyury T domain binds the T site oligonucleotide and that binding is competed by cold wild-type oligonucleotide but not by a mutated oligonucleotide. Panel b: Lanes 7–9 include 32P-labelled TCACACCT; lane 10 uses the indicated mutated version of this oligonucleotide. Note that the Brachyury T domain does not bind the mutated oligonucleotide. Panel c: Binding reaction using a 32P-labelled AC repeat oligonucleotide. Lanes 11–13 as panel a; lane 14 includes excess unlabelled probe; lane 15 includes excess of an unlabelled mutated probe; lane 16 is a ‘supershift’. Notice that the Brachyury T domain binds the AC repeat oligonucleotide weakly but that binding does not seem to be competed by cold wild-type oligonucleotide. The complex however is ‘supershifted’ using the Brachyury antibody. Panel d: Binding reactions using a 32P labelled motif that includes both the T site TCACACCT and an AC repeat. Lanes 17–20 show that Brachyury binds this oligonucleotide, and that binding is competed by cold wild-type oligonucleotide. Lanes 21 and 22 show that binding is not competed significantly by unlabelled oligonucleotides in which either motif is mutated. Lane 23 shows a ‘supershift’. Experiments in (a–d) were performed under identical conditions and exposed for the same times. (TIF) [file pone.0033346.s003.tif]

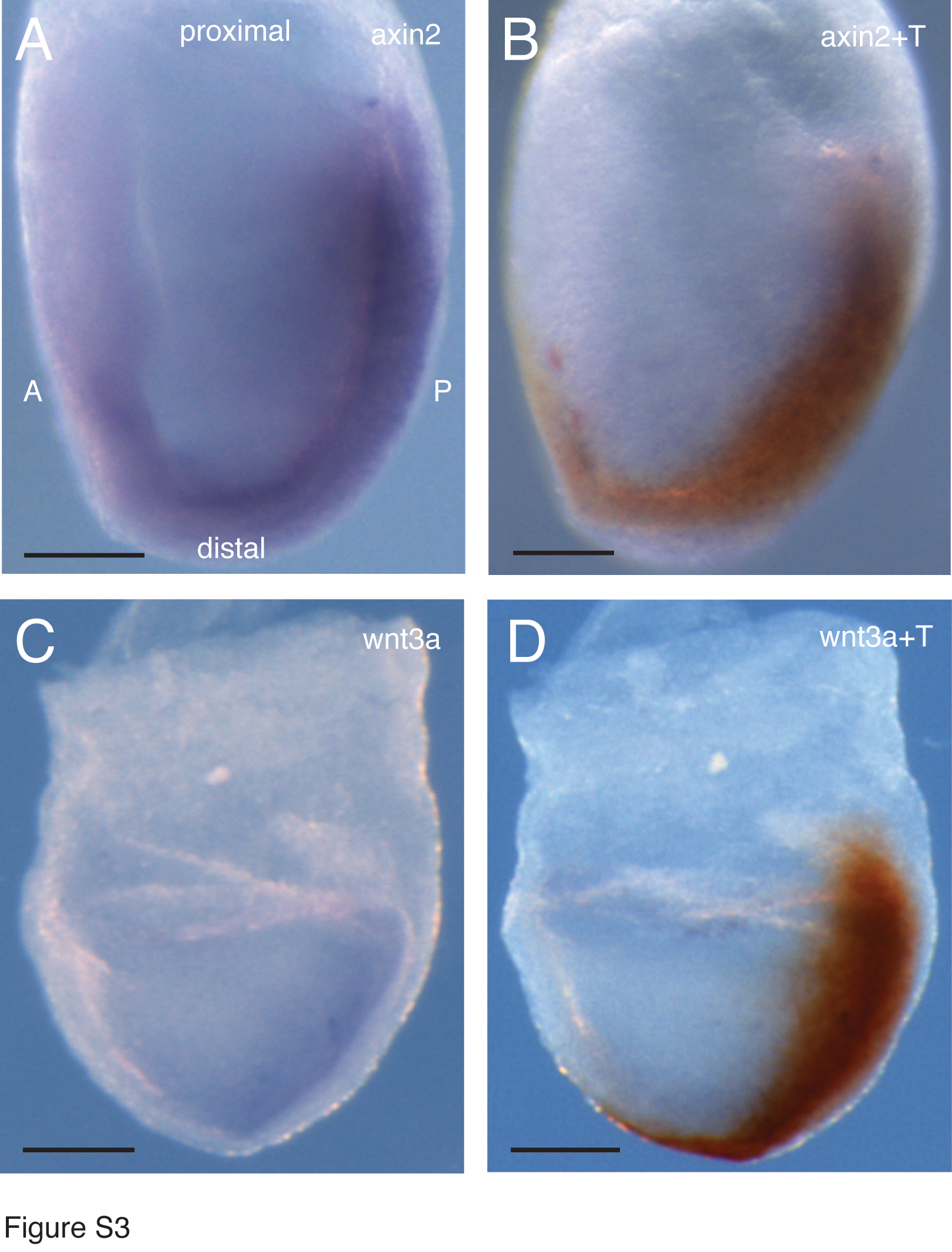

Supplement: Figure S4 — The expression domains of Brachyury , Wnt3a and Axin2 overlap in E7.75 mouse embryos. (A) Expression of Axin2 analysed using a fluorescein labelled antisense probe detected with NBT/BCIP (purple). (B) The embryo in (A) analysed using a digoxigenin labelled antisense Brachyury probe detected with INT/Mg phosphate (brown). (C) Expression of Wnt3a analysed using a fluorescein labelled antisense probe detected with NBT/BCIP (purple). (D) The embryo in (C) analysed using a digoxigenin labelled antisense Brachyury probe detected with INT/Mg phosphate (brown). All embryos orientated as in (A). Scale bars are 200 µm. (TIF) [file pone.0033346.s004.tif]

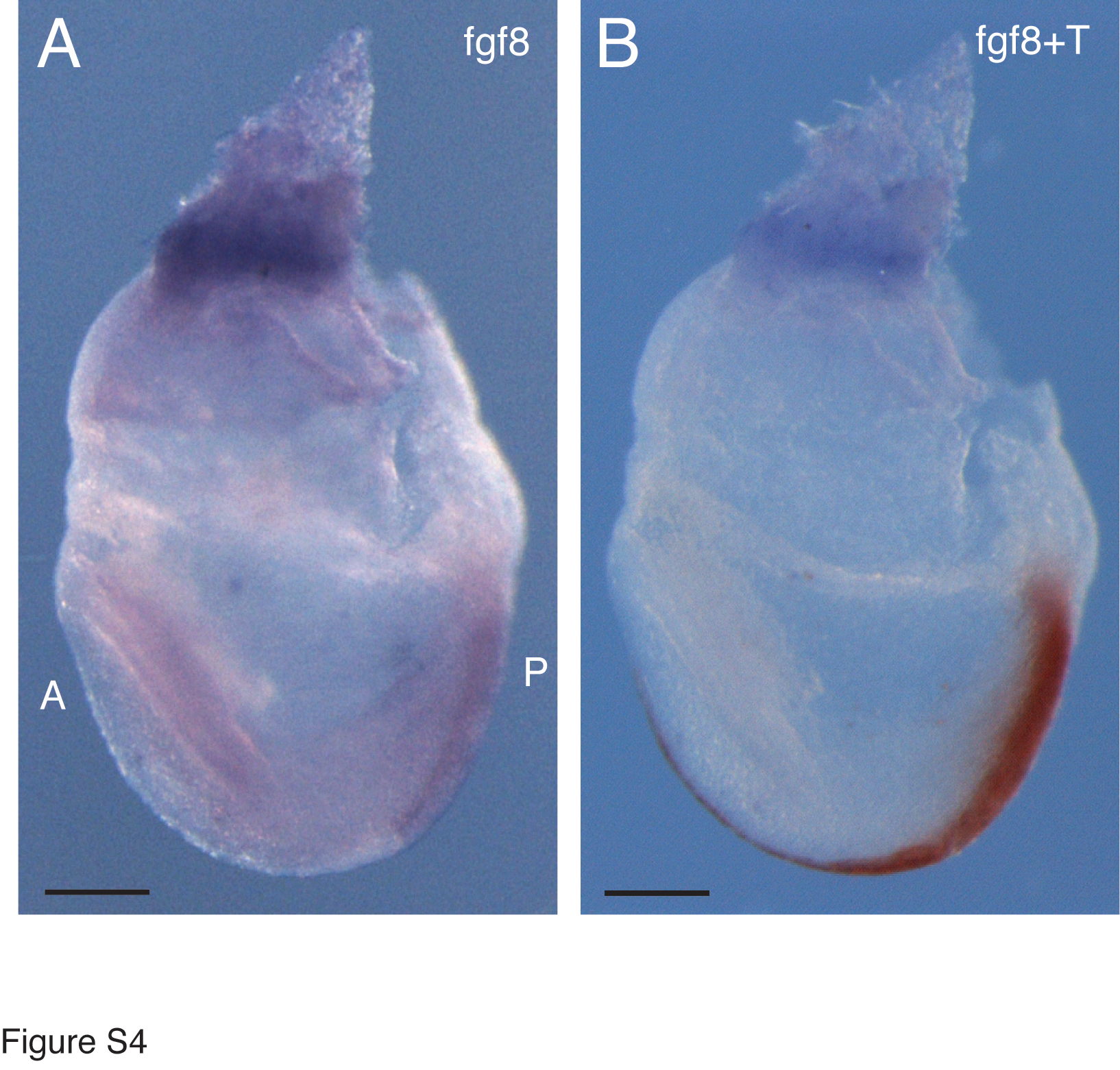

Supplement: Figure S5 — The expression domains of Brachyury and Fgf8 overlap in the primitive streak of E7.75 mouse embryos. (A). Expression of Fgf8 analysed using a fluorescein labelled antisense probe detected with NBT/BCIP (purple). (B). The embryo in (A) analysed using a digoxigenin labelled antisense Brachyury probe detected with INT/Mg phosphate (brown). Black bars are 200 µm. (TIF) [file pone.0033346.s005.tif]

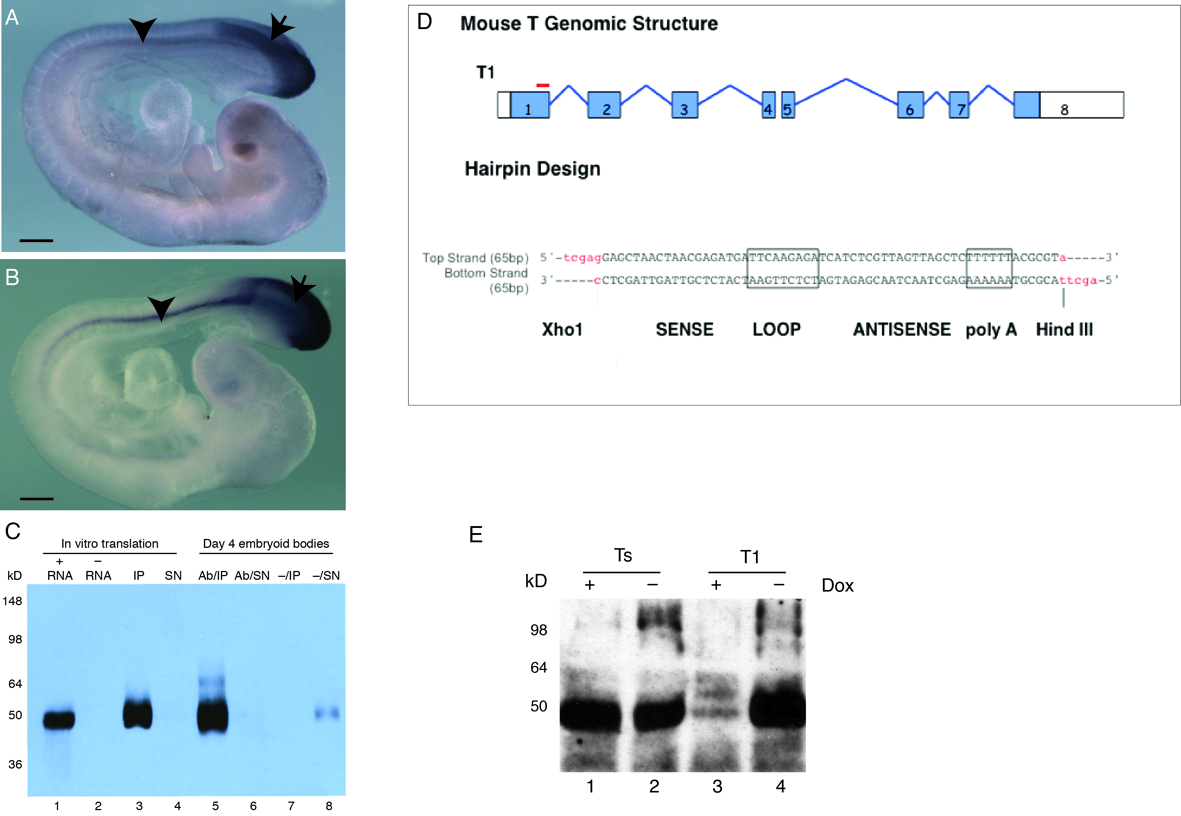

Supplement: Figure S6 — Verification of anti Brachyury antibody. (A) Immunohistochemistry of E9.5 embryo using Santa Cruz anti-human T C19 with nickel enhanced DAB substrate. Staining is present in the notochord (arrowhead), pre-somitic mesoderm (arrow) and tailbud. Staining was absent in controls in which primary or secondary antibodies were omitted. (B) Expression of Brachyury RNA in an E9.5 embryo studied by in situ hybridisation. Note similarity to (A). Bars in (A) and (B) represent 250 µm. (C) Western blot testing antibody specificity. Size markers are shown to the left. Lane 1: Mouse Brachyury reticulocyte lysate translation product; lane 2: unprogrammed reticulocyte lysate translation product; lane 3: Immunoprecipitated material derived from Brachyury reticulocyte lysate translation product; lane 4: Supernatant of immunoprecipitated material in lane 3; lane 5: Immunoprecipitated material derived from day 4 embryoid bodies; lane 6: Supernatant of immunoprecipitated material in lane 5; lane 7: Immunoprecipitated material derived from day 4 embryoid bodies, having omitted first antibody; lane 8: Supernatant of immunoprecipitated material in lane 7. All immunoprecipitations used Santa Cruz anti-T C19. Western blots used R&D Systems anti-T as a primary antibody and SantaCruz D anti-goat IgG HRP linked secondary antibody. (D) Strategy to create ES cell clones lacking Brachyury. Clones were created using 65 bp ShRNA duplexes targeting the first exon of Brachyury (T). Sequences were inserted into the XhoI/HindIII site of the pSingle ShRNA vector (Clontech) which includes a tetracyclin-controlled transcriptional repressor that in turn regulates the expression of the ShRNA sequence. Selection of stable lines is achieved by culture in G418 and induction of ShRNA expression occurs through addition of 1 µg/ml doxycycline. (E) Western blot analysis of day 5 embryoid body extracts from clones containing ShRNA constructs targeted to Brachyury exon 1 (T1) or a scrambled version of this sequence (Ts) [file pone.0033346.s006.tif]
