# Supplementary material for: Genomic Targets of Brachyury (T) in Differentiating Mouse Embryonic Stem Cells
Source: PLoS One. 2012 Mar 30;7(3):e33346. doi: 10.1371/journal.pone.0033346 (PMC3316570; doi:10.1371/journal.pone.0033346)
Supplement: Table S1 — Full gene list. (DOC) [file pone.0033346.s007.doc]

**Supplementary Table S1**

**Full Gene List**

| **Gene** | **Common Name** | **Chromosome** | **Peak**  **Position** |
| --- | --- | --- | --- |
| 0610040J01Rik | unknown | chr5 | promoter |
| 1110004B13Rik | Tmem107 | chr11 | promoter |
| 1110014K08Rik | D030012E24Rik | chr11 | promoter |
| 1110032A13Rik | RNA binding protein | chr18 | divergent |
| 1110059G10Rik | unknown | chr9 | inside |
| 1190002N15Rik | C3orf58 | chr9 | inside |
| 1200009I06Rik | 1600013K19Rik/sec6 | chr12 | inside |
| 1300014I06Rik | unknown | chr13 | promoter |
| 1700012A16Rik | unknown | chr1 | promoter |
| 1700029I08Rik | unknown | chr17 | promoter |
| 2310079F23Rik | unknown | chr5 | promoter |
| 2410146L05Rik | Ooep, oocyte expressed protein homolog (dog) | chr9 | promoter |
| 2610207I05Rik | 5430435M13Rik, C130002K18Rik, mKIAA0421, smg1 | chr7 | inside |
| 4732495E13Rik | transmembrane protein 184b | chr15 | inside |
| 4930404H21Rik | unknown/C16orf65 homolog | chr7 | inside |
| 4930408O21Rik | hypothetical PDZ domain containing protein Uqcrc2 | chr7 | promoter |
| 4930412F15Rik | unknown | chr4 | promoter |
| 4930441O14Rik | unknown | chr13 | inside |
| 4930504O13Rik | unknown | chr11 | promoter |
| 4930546H06Rik | B230206P06Rik | chr17 | inside |
| 4930556P03Rik | Bcdin3d BCDIN3 domain containing | chr15 | promoter |
| 4930578I06Rik | unknown | chr14 | inside |
| 5330431N19Rik | Dpcd, deleted in primary ciliary dyskinesia | chr19 | inside |
| 6330514A18Rik | polo-like kinase 5 | chr10 | inside |
| 6430573F11Rik | unknown | chr8 | inside |
| 9030425E11Rik | ACAM; ASP5; CLMP; AW557819 | chr9 | promoter |
| 9530068E07Rik | Keratinocytes-associated transmembrane protein 2 | chr11 | inside |
| A230056P14Rik | unknown | chr7 | inside |
| A630055G03Rik | LOC381034 | chr16 | promoter |
| A730017C20Rik | unknown function | chr18 | promoter |
| AA407270 | unknown | chr9 | promoter |
| Abcc2 | ATP-binding cassette, sub-family C (CFTR/MRP), member 2 C, Mrp2, multidrug resistance protein 2 | chr19 | promoter |
| Abhd1 | Abhydrolase domain containing 1,LABH-1, LABH1 | chr5 | promoter |
| Acly | ATP citrate lyase A730098H14Rik | chr11 | inside |
| Adam19 | A Disintegrin and metallopeptidase domain 19 (meltrin beta) | chr11 | inside |
| Adam24 | A Disintegrin and metallopeptidase domain 24 (testase 1) Dtgn5 | chr8 | promoter |
| Adam5 | A Disintegrin and metallopeptidase domain 5 tMDCII | chr8 | promoter |
| Adamts10 | ADAM metallopeptidase with thrombospondin type 1 motif 10, kuz, kuzbanian | chr17 | inside |
| Adar | adenosine deaminase, RNA-specific, ADAR1, mZaADAR | chr3 | inside |
| Aipl1 | aryl hydrocarbon receptor-interacting protein-like 1 | chr11 | divergent |
| Akap10 | A kinase (PRKA) anchor protein 10 | chr11 | promoter |
| Aldoc | aldolase 3, C isoform Aldo3, Aldolase C, Scrg2, zebrin II | chr11 | inside |
| Ankrd13d | ankyrin repeat domain 13 family, member D | chr19 | inside |
| Anxa7 | annexin A7 Anx7, synexin | chr14 | inside |
| Apoa2 | apolipoprotein A-II,Alp-2, Apoa-2, ApoA-II, Hdl-1 | chr1 | inside |
| Appbp1 | amyloid beta precursor protein binding protein1, Nae1, NEDD8 activating enzyme E1 | chr8 | divergent |
| Aqp11 | aquaporin 11 | chr7 | promoter |
| Aqp4 | aquaporin 4 | chr18 | promoter |
| Arhgef4 | rho/rac guanine nucleotide exchange factor (GEF) 4 | chr1 | promoter |
| Arid1a | AT rich interactive domain 1A (Swi1 like) BAF250a | chr4 | promoter |
| Atcay | ataxia, cerebellar, Cayman type homolog (human), BNIP-H, ji | chr10 | inside |
| Athl1 | ATH1, acid trehalase-like 1 (yeast) | chr7 | downstream |
| Atoh7 | atonal homolog 7 (Drosophila), Math5 | chr10 | promoter |
| Atp1a3 | ATPase, Na+/K+ transporting, alpha 3 polypeptide, Atpa-2 | chr7 | promoter |
| Atp6v1d | ATPase H+ transporting lysosomal V1 subunit D, lysosomal 34kDa, VATD, Vma8 | chr12 | inside |
| AU041783 | Afap1l2, actin filament associated protein 1-like 2 | chr19 | inside |
| AW544981 | Mouse E7.5 Extraembryonic cDNA Library | chr15 | promoter |
| Axin2 | Axin2/axil/conductin | chr11 | promoter |
| B230218O03 | maybe related Homo sapiens neuroplastoma apoptosis-related RNA-binding protein (CUGBP2) | chr2 | promoter |
| B4galnt2 | beta-1,4-N-acetyl-galactosaminyl transferase 2 Dlb-1, Dlb1, Galgt2 | chr11 | inside |
| Bapx1 | NK3 homeobox 2 Bapx1, Nkx-3.2, Nkx3-2 | chr5 | promoter |
| BC002017 | phospholipid scramblase 1Plscr1 MmTRA1a, MmTRA1b, MuPLSCR2, NOR1, Tras1/2 | chr9 | Unknown |
| BC017647 | unknown | chr11 | inside |
| BC019143 | 1300010F03Rik | chr14 | Unknown |
| BC035295 | Csnrp2 cysteine-serine-rich nuclear protein 2 | chr15 | promoter |
| BC048651 | LOC330277, NYD-SP18 | chr6 | promoter |
| BC052496 | Grap2,GRB2-related adaptor protein 2, Gads, GRAP-2, Grb2-related adaptor downstream of Sch, GRB2L, GrbX, Grf40, GRID, GrpL, Mona, P38 | chr15 | Unknown |
| BC054059 | SMAF1likely orthologue of H. sapiens small adipocyte factor 1 (SMAF1) | chr2 | inside |
| BC072639 | 2010300C02Rik | chr1 | unknown |
| BC100404 | Cpm, carboxypeptidase M | chr10 | unknown |
| Bcl6 | B-cell leukemia/lymphoma 6 Bcl5 | chr16 | promoter |
| Blr1 | burkitt lymphoma receptor 1 Blr1, CXCR-5, Gpcr6 | chr9 | promoter |
| Bmp1 | bone morphogenetic protein 1 | chr14 | inside |
| Boc | biregional cell adhesion molecule-related/down-regulated by oncogenes (Cdon) binding protein | chr16 | inside |
| C030030A07Rik | transmembrane protein 72 | chr6 | inside |
| C1qtnf6 | C1q and tumor necrosis factor related protein 6, CTRP6 | chr15 | inside |
| C230095G01Rik | unknown | chr6 | promoter |
| C78339 | unknown | chr13 | promoter |
| C79407 | DNA binding protein | chr12 | promoter |
| Cables2 | Cdk5 and Abl enzyme substrate 2 ik3-2 | chr2 | promoter |
| Camk2a | calcium/calmodulin-dependent protein kinase II alpha alpha-CaMKI | chr18 | inside |
| Cant1 | C calcium activated nucleotidase 1, SCAN-1, Shapy, Apy1h | chr11 | promoter |
| Capns1 | calpain, small subunit 1, Capa4, Capn4 | chr7 | promoter |
| Car10 | CA-RP X | chr11 | inside |
| Carhsp1 | calcium regulated heat stable protein 1 | chr16 | promoter |
| Cbln3 | cerebellin 3 precursor protein | chr14 | inside |
| Ccdc52 | coiled-coil domain containing 52 | chr16 | inside |
| Ccdc85a | coiled-coil domain containing 85A | chr11 | promoter |
| Ccl1 | chemokine (C-C motif) ligand 1, Scya1, Tca-3 | chr11 | inside |
| Cd300e | CD300e antigen Cd300le, Clm2, Trem5 | chr11 | promoter |
| Cdc42ep5 | CDC42 effector protein (Rho GTPase binding) 5, Borg3, CEP5 | chr7 | promoter |
| Cdk9 | cyclin-dependent kinase 9 (CDC2-related kinase) PITALRE | chr2 | inside |
| Cebpa | CCAAT/enhancer binding protein (C/EBP), alpha | chr7 | promoter |
| Ces6 | carboxylesterase 6 9130231C15Rik | chr8 | promoter |
| Chn2 | chimerin (chimaerin) 2, Rho GTPase-activating protein 3 | chr6 | promoter |
| Chrm2 | cholinergic receptor, muscarinic 2, cardiac AChR M2, M2 | chr6 | promoter |
| Chrnb1 | cholinergic receptor, nicotinic, beta polypeptide 1 (muscle) Achr-2, Acrb | chr11 | inside |
| Chst8 | carbohydrate (N-acetylgalactosamine 4-0) sulfotransferase 8 GalNAc4ST-1 | chr7 | promoter |
| Cit | Citron, Cit-k, citron kinase, citron-N, CRIK, CRIK-SK | chr5 | inside |
| Clnk | cytokine-dependent hematopoietic cell l linker MIST D5Dmo5 | chr5 | inside |
| Cml4 | camello-like 4, Nat8 N-acetyltransferase 8 (GCN5-related, putative) | chr6 | promoter |
| Cmtm2b | CKLF-like MARVEL transmembrane domain containing 2B | chr8 | inside |
| Cnga3 | cyclic nucleotide gated channel alpha 3 CNG3 | chr1 | promoter |
| Cnn1 | calponin 1 calponin h1, CN, CnnI | chr9 | promoter |
| Coq10b | coenzyme Q10 homolog B (S. cerevisiae) | chr1 | promoter |
| Coq2 | coenzyme Q2 homolog, prenyltransferase (yeast) | chr5 | promoter |
| Cryba2 | crystallin beta A2 | chr1 | inside |
| Crygn | crystalline gamma N | chr5 | inside |
| Ctla4 | cytotoxic T-lymphocyte-associated protein 4, Cd152, Ctla-4, Ly-56 | chr1 | promoter |
| Ctnnb1 | catenin (cadherin associated protein), beta 1 Catnb, Mesc | chr9 | promoter |
| Ctrc | chymotrypsin C (caldecrin) ELA4, Elastase iv | chr4 | inside |
| Cugbp2 | CUG triplet repeat, RNA binding protein 2, ETR-3, Napor-2 | chr2 | promoter |
| Cygb | cytoglobin, Staap | chr11 | promoter |
| D030011O10Rik | Dennd5b, DENN/MADD domain containing 5B | chr6 | promoter |
| D130058I21Rik | Smtnl2 smoothelin-like 2 | chr11 | promoter |
| D630039A03Rik | unknown | chr4 | inside |
| Ddef1 | development and differentiation enhancing Asap1 | chr15 | inside |
| Defb27 | beta defensin 27,similar to beta defensin 123 predicted gene | chr2 | inside |
| Dhrs3 | dehydrogenase/reductase (SDR family) member 3 | chr4 | inside |
| Diras2 | DIRAS family, GTP-binding RAS-like 2 | chr13 | promoter |
| Dkk1 | dickkopf homolog 1 (Xenopus laevis) | chr19 | inside |
| Dlx5 | distal-less homeobox 5 | chr6 | inside |
| Dnase1l3 | deoxyribonuclease 1-like 3 | chr14 | divergent |
| Dpp4 | dipeptidylpeptidase 4, CD26, THAM | chr2 | promoter |
| Dscr1l1 | regulator of calcineurin 2,Csp2, Dscr1l1, MCIP2, ZAKI-4, new symbol Rcan2 | chr17 | promoter |
| Dvl3 | dishevelled 3, dsh homolog (Drosophila) | chr16 | inside |
| Dync1li1 | dynein cytoplasmic 1 light intermediate chain 1, Dnclic1, LIC-1 | chr9 | inside |
| Dync2li1 | dynein cytoplasmic 2 light intermediate chain 1, D2lic, LIC3, mD2LIC | chr17 | promoter |
| E130309D14Rik | unknown | chr11 | downstream |
| E430004N04Rik | Themis, thymocyte selection associated, Gasp, Tsepa | chr10 | promoter |
| Ebf1 | early B-cell factor 1 O/E-1, Olf-1, Olf1 | chr11 | inside |
| Ebf2 | early B-cell factor 2 D14Ggc1e, Mmot1, O/E-3 | chr14 | promoter |
| EG574403 | predicted gene, conserved | chr11 | promoter |
| Elavl4 | ELAV (embryonic lethal, abnormal vision, Drosophila)-like 4 (Hu antigen D) | chr4 | promoter |
| Elmod1 | ELMO domain containing 1 | chr9 | promoter |
| Epb4.1l4a | erythrocyte protein band 4.1-like 4a /NBL4 | chr18 | inside |
| Epb4.9 | dematin, erythrocyte protein band 4.9 | chr14 | promoter |
| Ephx1 | epoxide hydrolase 1, microsomal Eph-1, Eph1 | chr1 | promoter |
| Erg | avian erythroblastosis virus E-26 (v-ets) oncogene related | chr16 | promoter |
| Etv1 | ets variant gene 1, ER81, Etsrp81 | chr12 | promoter |
| Eva1 | Mpzl2 myelin protein zero-like 2 | chr9 | inside |
| Fabp1 | fatty acid binding protein 1, liver Fabpl, L-FABP, MGC:13855 | chr6 | promoter |
| Farp1 | FERM, RhoGEF (Arhgef) and pleckstrin domain protein 1 (chondrocyte-derived) Cdep | chr14 | inside |
| Fbxl10 | F-box and leucine-rich repeat protein 10 Cxxc2, Jhdm1b, KDM2B, JEMMA (Jumonji domain, EMSY-interactor, methyltransferase motif) | chr5 | inside |
| Fev | FEV (ETS oncogene family) mPet-1, Pet1 Fifth Ewing Variant | chr1 | promoter |
| Fgf8 | Fibroblast growth factor 8, Aigf | chr19 | promoter |
| Foxa2 | Forkhead box A2 Hnf-3b, HNF3-beta, Hnf3b, HNF3beta, Tcf-3b, Tcf3b | chr2 | promoter |
| Foxe1 | Forkhead box E1 (thyroid transcription factor 2), Titf2 | chr4 | promoter |
| Foxi2 | Forkhead box I2 | chr7 | promoter |
| Foxn1 | Forkhead box N1, Hfh11, whn | chr11 | promoter |
| Fut8 | fucosyltransferase 8, alpha (1,6) fucosyltransferase | chr12 | promoter |
| Fxyd6 | FXYD domain-containing ion transport regulator 6, Php | chr9 | promoter |
| Fyb | FYN binding protein, ADAP, FYB-120/130 | chr15 | promoter |
| Gabra1 | gamma-aminobutyric acid (GABA-A) receptor, subunit alpha 1 | chr11 | promoter |
| Gabra2 | gamma-aminobutyric acid (GABA) A receptor, alpha 2 | chr5 | inside |
| Gabrb2 | gamma-aminobutyric acid ( GABA) A receptor, beta 2 | chr11 | promoter |
| Gcnt2 | glucosaminyl (N-acetyl) transferase 2, I-branching enzyme, IGnTA, IGnTB, IGnTC | chr13 | promoter |
| Gdf5 | growth differentiation factor 5 (cartilage-derived morphogenetic protein-1), CDMP-1 | chr2 | inside |
| Gdpd4 | glycerophosphodiester phosphodiesterase domain containing 4 | chr7 | promoter |
| Gfra3 | glial cell line derived neurotrophic factor family receptor alpha 3 | chr18 | promoter |
| Glt8d1 | glycosyltransferase 8 domain containing 1 | chr14 | inside |
| Glt8d3 | glycosyltransferase 8 domain containing 3 | chr15 | promoter |
| Gm879 | gene model 879 | chr11 | promoter |
| Gnaq | guanine nucleotide binding protein, alpha q polypeptide Dsk1, Dsk10, G alpha q, Gq | chr19 | promoter |
| Gnaz | guanine nucleotide binding protein, alpha z subunit Gz | chr10 | inside |
| Gnb2l1 | RACK1 receptor for activated kinase C1 | chr11 | promoter |
| Gsg1 | germ cell-specific gene 1 | chr6 | promoter |
| Gstm7 | glutathione S-transferase, mu 7, Cd203c, GSTm2 muscle | chr3 | inside |
| H2afy2 | H2A histone family, member Y2 macroH2A2 | chr10 | promoter |
| Hcrtr1 | hypocretin (orexin) receptor 1OX1R | chr4 | promoter |
| Hdgfrp2 | hepatoma-derived growth factor, related protein 2 HRP-2 | chr17 | INSIDE |
| Hnrpl | Hnrnpl heterogeneous nuclear ribonucleoprotein L, Hnrpl | chr7 | promoter |
| Hnrpul1 | heterogeneous nuclear ribonucleoprotein U-like 1, E1B-AP5 | chr7 | inside |
| Hoxa13 | homeo box A13 Hox-1.10 | chr6 | promoter |
| Hoxa3 | homeo box A3 | chr6 | divergent |
| Hsd17b3 | hydroxysteroid (17-beta) dehydrogenase 3 17(beta)HSD type 3 | chr13 | inside |
| I830134H01Rik | unknown | chr19 | inside |
| Ica1 | islet cell autoantigen 169kDa, ICA69 | chr6 | inside |
| Id4 | inhibitor of DNA binding 4 Idb4 | chr13 | promoter |
| Igsf11 | immunoglobulin superfamily, member 11 | chr16 | promoter |
| Il1r1 | interleukin 1 receptor, type i | chr1 | inside |
| Il21 | Interleukin-21 | chr3 | promoter |
| Il6 | interleukin 6 | chr5 | promoter |
| Irx6 | Iroquois related homeobox 6 (Drosophila) | chr8 | inside |
| Junb | Jun-B oncogene/AP-1 activator protein 1 | chr8 | divergent |
| Jup | junction plakoglobin, gamma-catenin, PG, plakoglobin | chr11 | promoter |
| Kcnd3 | potassium voltage-gated channel, member 3 potassium channel Kv4.3L | chr3 | inside |
| Kcnn4 | potassium intermediate/small conductance calcium-activated channel, subfamily N, member 4 IK1, mIKCa1, SK4 | chr7 | promoter |
| Kctd13 | potassium channel tetramerisation domain containing 13, Poldip1 | chr7 | promoter |
| Kif23 | kinesin family member 23, C87313, CHO1, Knsl5, MKLP-1, MKLP1 | chr9 | inside |
| Krt16 | keratin 16, K16, Krt1-16 | chr11 | promoter |
| Krt5 | keratin 5, K5, Krt2-5, Tfip8 | chr15 | promoter |
| Krt8 | Card2, cytokeratin 8, EndoA, K8, Krt-2.8, Krt2-8 | chr15 | promoter |
| Lama4 | laminin, alpha 4 | chr10 | promoter |
| Lancl1 | LanC (bacterial lantibiotic synthetase component C)-like 1, LanC-like protein 1, p40 | chr1 | promoter |
| Laptm5/E3 | lysosomal-associated protein transmembrane 5 Retinoic acid-inducible E3 protein | chr4 | promoter |
| Large | like-glycosyltransferase BPFD#36, enr, fg, froggy | chr8 | promoter |
| Lctl | lactase-like, KLPH, klotho gamma,Klotho/lactase-phlorizin hydrolase- related protein | chr9 | promoter |
| Leprotl1 | leptin receptor overlapping transcript-like 1 | chr8 | divergent |
| Lhfpl2 | lipoma HMGIC fusion partner-like 2 | chr13 | promoter |
| Lhx5/Lim2 | LIM homeobox protein 5 | chr5 | inside |
| Lif | leukemia inhibitory factor | chr11 | promoter |
| Lifr | leukemia inhibitory factor receptor, soluble differentiation-stimulating factor receptor | chr15 | promoter |
| Lmx1b | LIM homeobox transcription factor 1 beta LMX1.2 | chr2 | inside |
| Lphn3 | latrophilin 3, LEC3, lectomedin 3 | chr5 | promoter |
| Lrig1 | leucine-rich repeats and immunoglobulin-like domains 1 Img, LIG-1 | chr6 | promoter |
| Lrrc4c | leucine rich repeat containing 4C, netrin g1 ligand | chr2 | promoter |
| Map2k5 | mitogen activated protein kinase kinase 5 MAPK/ERK kinase 5, Mapkk5, MEK5 | chr9 | promoter |
| Map3k11 | mitogen activated protein kinase kinase kinase 11 2610017K16Rik, Mlk3, PTK1 | chr19 | inside |
| Map3k2 | mitogen activated protein kinase kinase kinase 2, MEK kinase 2, Mekk2 | chr18 | promoter |
| Map3k3 | mitogen activated protein kinase kinase kinase 3 MAPKKK3, Mekk3 | chr11 | promoter |
| Mapre2/EB1/EB2 | microtubule-associated protein, RP/EB family, member 2 C820009F03Rik, EB2, RP1 | chr18 | promoter |
| Marcks | myristoylated alanine rich protein kinase C substrate Macs | chr10 | inside |
| Marcksl1 | MARCKS-like 1, F52, MacMARCKS, Macs-2, Macs-3, Macs2, Macs3, Mlp, Mrp | chr4 | promoter |
| Mbp | myelin basic protein golli-mbp, Hmbpr | chr18 | inside |
| Med4 | mediator of RNA polymerase II transcription, subunit 4 homolog (yeast), DRIP36, HSPC126, p36 TRAP/SMCC/PC2 subunit, TRAP36, Vdrip | chr14 | inside |
| Meis1 | Meis homeobox 1 | chr11 | promoter |
| MGC117608 | possible psuedogene similar to mitochondrial protein L32 | chr6 | inside |
| Mical1 | microtubule associated monoxygenase, calponin and LIM domain containing, Nical | chr10 | promoter |
| Morn2 | MORN repeat containing 2 Mopt | chr17 | promoter |
| Mov10 | Moloney leukemia virus 10 | chr3 | promoter |
| Mrpl14 | mitochondrial ribosomal protein L14, MRP-L32, Rpml32 | chr17 | inside |
| Mrps23 | mitochondrial ribosomal protein S23, Rpms23 | chr11 | promoter |
| Msgn1 | mesogenin 1 | chr12 | downstream |
| Msi2 | Musashi homolog 2 (Drosophila) msi2h, Musashi 2 | chr11 | promoter |
| Mttp | microsomal triglyceride transfer protein | chr3 | inside |
| Nat3 | N-acetyltransferase 3 | chr8 | promoter |
| Ndn | necdin Peg6 | chr7 | promoter |
| Nedd4l | neural precursor cell expressed, developmentally down-regulated gene 4-like 1, Nedd4b | chr18 | inside |
| Neu3 | neuraminidase 3 ganglioside sialidase, membrane sialidase | chr7 | inside |
| Ngb | neuroglobin | chr12 | inside |
| Nhlrc1 | NHL repeat containing 1, EPM2B, Malin | chr13 | promoter |
| Nhp2l1 | NHP2 non-histone chromosome protein 2-like 1(S. cerevisiae), FA-1, Fertilization antigen-1, Fta1, Ssfa1 | chr15 | promoter |
| Nkx2-6 | NK2 transcription factor related, locus 6 tinman, Tix | chr14 | inside |
| Nlrp4b | NLR family, pyrin domain containing 4B | chr7 | promoter |
| Npffr2 | neuropeptide FF receptor 2 Gpr74, NPFF2 | chr5 | promoter |
| Nsl1 | NSL1, MIND kinetochore complex component, homolog (S. cerevisiae) | chr1 | promoter |
| Nudt16 | nudix (nucleoside diphosphate linked moiety X)-type motif 16 | chr9 | promoter |
| Oas1f | 2'-5' oligoadenylate synthetase 1F | chr5 | inside |
| Olfr1026 | olfactory receptor 1026 GA_x6K02T2Q125-47402610-47403533, MOR196-4 | chr2 | promoter |
| Olfr1388 | olfactory receptor 1388 GA_x6K02T2QP88-5991012-5990077, MOR256-28 | chr11 | promoter |
| Olfr209 | olfactory receptor 209 GA_x54KRFPKG5P-55590495-55589578, MOR182-6 | chr16 | promoter |
| Olfr473 | olfactory receptor 473 GA_x6K02T2PBJ9-10262759-10263691, MOR204-4 | chr7 | promoter |
| Olfr702 | olfactory receptor 702 GA_x6K02T2PBJ9-9202245-9201289, MOR260-4 | chr7 | promoter |
| Olfr71 | olfactory receptor 71 GA_x6K02T2N78B-16230286-16231224, mOR17, MOR262-4 | chr4 | promoter |
| Olfr907 | olfactory receptor 907 GA_x6K02T2PVTD-32204729-32205661, MOR165-5 | chr9 | promoter |
| Olfr934 | olfactory receptor 934 GA_x6K02T2PVTD-32678895-32677963, MOR224-6 | chr9 | promoter |
| Olfr95 | olfactory receptor 95 GA_x6K02T2PSCP-1651760-1650822, MOR263-6 | chr17 | downstream |
| Otop2 | otopetrin 2 | chr11 | inside |
| Ovgp1 | oviductal glycoprotein 1, 120kDa, Chit5, MOGP, muc 9, OGP, Ovgp1, oviductin | chr3 | promoter |
| Oxr1 | oxidation resistance, C7, C7B | chr15 | promoter |
| Pabpn1 | poly(A) binding protein, nuclear 1PAB2, Pabp3, poly(A) binding protein II | chr14 | promoter |
| Palmd | palmdelphin, PALML | chr3 | inside |
| Park7 | Parkinson disease (autosomal recessive, early onset) 7, DJ-1, Dj1 | chr4 | divergent |
| Parvg | parvin, gamma | chr15 | promoter |
| Pax2 | paired box gene 2 | chr19 | promoter |
| Pax3 | paired box gene 3 | chr1 | inside |
| Pax5 | paired box gene 5, B cell-specific activator protein EBB-1 BSAP | chr4 | inside |
| Pcdha7 | protocadherin alpha 7 Crnr4 | chr18 | promoter |
| Pck2 | phosphoenolpyruvate carboxykinase 2 (mitochondrial) | chr14 | promoter |
| Pcolce2 | procollagen C-endopeptidase enhancer 2, Pcpe2 | chr9 | promoter |
| Pde2a | phosphodiesterase 2A, cGMP-stimulated | chr7 | promoter |
| Pde7b | phosphodiesterase 7B | chr10 | inside |
| Pdpk1 | 3-phosphoinositide dependent protein kinase-1 Pdk1, Pkb kinase | chr17 | inside |
| Pdss1 | prenyl (solanesyl) diphosphate synthase, subunit 1, mSPS1, Tprt | chr2 | promoter |
| Pdx1 | pancreatic and duodenal homeobox 1,IDX-1, IPF-1, Ipf1, Mody4, pdx-1, STF-1 | chr5 | promoter |
| Pelp1 | proline, glutamic acid and leucine rich protein 1 | chr11 | promoter |
| Pfn4 | profilin family, member 4 | chr12 | inside |
| Pigc | phosphatidylinositol glycan anchor biosynthesis, class C | chr1 | promoter |
| Pigk | phosphatidylinositol glycan anchor biosynthesis, class K | chr3 | promoter |
| Pitpnc1 | phosphatidylinositol transfer protein, cytoplasmic 1, RDGB-BETA, RDGBB1 | chr11 | divergent |
| Pogk | pogo transposable element with KRAB domain, BASS2 | chr1 | promoter |
| Pold3 | polymerase (DNA-directed), delta 3, accessory subunit, C85233, GC12, P66, P68 | chr7 | promoter |
| Ppargc1a | peroxisome proliferative activated receptor, gamma, coactivator 1 alpha, Pgc-1alpha, Pgc-1alphaa, Pgc1, Pgco1, PPAR Gamma Coactivator-1 | chr5 | promoter |
| Ppargc1b | peroxisome proliferative activated receptor, gamma coactivator 1 beta, Perc, PGC-1beta/ERRL1 | chr18 | inside |
| Ppm1m | protein phosphatase 1M | chr9 | divergent |
| Ppp2r2b | protein phosphatase 2 (formerly 2A) regulatory subunit B (PR 52), beta isoform PP2A-PR55B, PR55-BETA, SCA12 | chr18 | promoter |
| Pqlc3 | PQ loop repeat containing | chr12 | promoter |
| Prcp | prolylcarboxypeptidase (angiotensinase C) | chr7 | promoter |
| Prkag3 | protein kinase, AMP-activated, gamma 3 non-catatlytic subunit, AMPKg3L, AMPKg3S | chr1 | promoter |
| Prlpa | prolactin family 4, subfamily a, member 1 PLP-A, Prlpa | chr13 | promoter |
| Prune | prune homolog (Drosophila) Prune-M1 | chr3 | inside |
| Psap | prosaposin SGP-1 | chr10 | inside |
| Psmd2 | proteasome (prosome, macropain) 26S subunit, non-ATPase, TEG-190, Tex190 | chr16 | promoter |
| Ptgir | prostaglandin i receptor (ip) IP, prostacyclin receptor | chr7 | promoter |
| Ptk2b | PTK2 protein tyrosine kinase 2 beta, CAKbeta, calcium-dependent tyrosine kinase, cellular adhesion kinase beta, proline-rich tyrosine kinase 2, PYK2, Raftk, related adhesion focal tyrosine kinase | chr14 | inside |
| Ptn | Pleiotrophin, Osf1, heparin-binding growth factor 8, HBGF-8, HB-GAM, heparin-binding growth-associated molecule | chr6 | inside |
| Pyy | peptide YY | chr11 | promoter |
| Qpct | glutaminyl-peptide cyclotransferase (glutaminyl cyclase) | chr17 | promoter |
| R74862 | expressed sequence R74862 | chr7 | promoter |
| Rab11fip4 | RAB11 family interacting protein 4 (class II), RAB11-FIP4 | chr11 | inside |
| Rab15 | RAB15, member RAS oncogene family | chr12 | inside |
| Rab17 | RAB17, member RAS oncogene family | chr1 | promoter |
| Rab5b | RAB5B, member RAS oncogene family | chr10 | promoter |
| Rab6b | RAB6B, member RAS oncogene family | chr9 | promoter |
| Ralgps2 | Ral GEF with PH domain and SH3 binding motif | chr1 | inside |
| Rbed1 | RNA binding motif and ELMO domain 1, ELMOD3, RBM29 | chr6 | inside |
| Rbp1 | retinol binding protein 1, cellular, Crbp, CRBPI | chr9 | promoter |
| Rgs2 | regulator of G-protein signaling 2 GOS8 | chr1 | inside |
| Rhou | Ras homolog gene family, member U, Arhu, CDC42L1, mG28K, WRCH1 | chr8 | promoter |
| Ripk5 | receptor interacting protein kinase 5 dusty protein kinase | chr1 | inside |
| Rnf207 | ring finger protein 207 | chr4 | divergent |
| Rnf26 | ring finger protein 26 | chr9 | promoter |
| Robo4 | roundabout homolog 4 (Drosophila), Magic roundabout | chr9 | first intron |
| Rpl36 | ribosomal protein L36 | chr17 | inside |
| Rsl1 | regulator of sex limited protein 1 rslcan-9 | chr13 | promoter |
| Rttn | rotatin (no turning) | chr18 | promoter |
| Rufy3 | RUN and FYVE domain containing 3, mKIAA0871, Rpipx | chr5 | inside |
| Sart2 | dermatan sulfate epimerase new symbol Dse | chr10 | promoter |
| Schip1 | schwannomin interacting protein 1 merlin | chr3 | promoter |
| Scml4 | sex comb on midleg-like 4 (Drosophila) | chr10 | promoter |
| Scrg1 | scrapie responsive gene 1 | chr8 | inside |
| Sec22a | SEC22 vesicle trafficking protein-like A (S. cerevisiae), Sec22l2 | chr16 | promoter |
| Sema4a | sema domain, immunoglobulin domain (Ig), transmembrane domain (TM) and short cytoplasmic domain, (semaphorin) 4A, Semab, SemB | chr3 | promoter |
| Serpinb1c | serine (or cysteine) peptidase inhibitor, clade B, member 1c EIC, ovalbumin | chr13 | promoter |
| Serpinb6c | serine (or cysteine) peptidase inhibitor, clade B, member 6c ovalbumin, Spi3C, SPIC | chr13 | promoter |
| Sertad2 | SERTA domain containing 2, SEI-2, Sei2, Trip-Br2 | chr11 | promoter |
| Sh3px3 | SH3 and PX domain containing 3 sorting nexin | chr9 | divergent |
| Slc16a6 | solute carrier family 16 (monocarboxylic acid transporters), member 6 | chr11 | promoter |
| Slc17a5 | solute carrier family 17 (anion/sugar transporter), member 5 | chr9 | promoter |
| Slc25a29 | solute carrier family 25 (mitochondrial carrier, palmitoylcarnitine transporter) 29 | chr12 | promoter |
| Slc25a37 | solute carrier family 25, member 37, Frascati, Mfrn, mitoferrin, Mscp | chr14 | promoter |
| Slc30a7 | solute carrier family 30 (zinc transporter), member 7, ZnT-7, ZnT7 | chr3 | inside |
| Slc37a2 | solute carrier family 37 (glycerol-3-phosphate transporter), member cI-2, G3PP, Slc37a1 | chr9 | inside |
| Slc41a3 | solute carrier family 41, member 3, SLC41A1-L2 | chr6 | promoter |
| Slc5a3 | solute carrier family 5 (inositol transporters), member 3 Smit1 | chr16 | promoter |
| Slc5a6 | solute carrier family 5 (sodium-dependent vitamin transporter), member 6 | chr5 | inside |
| Slc5a8 | solute carrier family 5 (iodide transporter), member 8 MGC:19357, SMCT | chr10 | promoter |
| Slu7 | SLU7 splicing factor homolog (S. cerevisiae) | chr11 | inside |
| Smarca2 | SWI/SNF related, matrix associated, actin dependent regulator of chromatin, subfamily a, member 2 brm, Snf2l2 | chr19 | promoter |
| Snai2 | snail homolog 2 (Drosophila), Slug, Slugh, Snail2 | chr16 | inside |
| Snx26 | sorting nexin 26 Tcgap | chr7 | promoter |
| Sppl3 | signal peptide peptidase 3, Usmg3 | chr5 | promoter |
| Srrp | serine-arginine repressor protein | chr4 | promoter |
| Sstr3 | somatostatin receptor 3, Smstr3, sst3 | chr15 | inside |
| St6galnac1 | ST6 (alpha-N-acetyl-neuraminyl-2,3-beta-galactosyl-1,3)-N-acetylgalactosaminide alpha-2,6-sialyltransferase 1, Siat7a, ST6GalNAc I | chr11 | promoter |
| St8sia3 | ST8 alpha-N-acetyl-neuraminide alpha-2, 8-sialyltransferase 3 Siat8c, ST8SiaIII | chr18 | inside |
| Stk38 | serine/threonine kinase 38, Ndr1 | chr17 | promoter |
| Stmn1 | stathmin 1, Lag, Lap18, leukemia associated phosphoprotein p18, metablastin, oncoprotein 18, op18, p18, p19, pig, PP17, PP18, PR22, prosolin, SMN | chr4 | inside |
| Stra13 | stimulated by retinoic acid 13 | chr11 | downstream |
| Stra6 | stimulated by retinoic acid gene 6 | chr9 | promoter |
| Stxbp1 | syntaxin binding protein 1, Munc-18a, N-sec1, Rb-sec1, Sxtbp1, Unc18h | chr2 | inside |
| Sumo2 | SMT3 suppressor of mif two 3 homolog 2 (yeast), Smt3b, Smt3h2, SUMO-2 | chr11 | inside |
| Syk | spleen tyrosine kinase | chr13 | promoter |
| Synpr | synaptoporin | chr14 | inside |
| Syt1 | synaptotagmin I | chr10 | inside |
| Tbc1d21 | TBC1 domain family, member 21 | chr9 | promoter |
| Tbc1d9b | TBC1 domain family, member 9B | chr11 | inside |
| Tbl1xr1 | transducin (beta)-like 1X-linked receptor 1, C21, DC42, Ira1, TBLR1 | chr3 | promoter |
| Tcf19 | transcription factor 19 | chr17 | inside |
| Tcl1b2 | T-cell leukemia/lymphoma 1B, 2 | chr12 | inside |
| Tfg | Trk-fused gene | chr16 | promoter |
| Tgfa | Transforming growth factor alpha | chr6 | promoter |
| Tiprl | TIP41, TOR signalling pathway regulator-like (S. cerevisiae) | chr1 | promoter |
| Tmbim4 | transmembrane BAX inhibitor motif containing 4 | chr10 | inside |
| Tmcc2 | transmembrane and coiled-coil domains 2 | chr1 | inside |
| Tmem10 | transmembrane protein10, Opalin, oligodendrocytic myelin paranodal inner loop protein | chr19 | promoter |
| Tmprss11e | transmembrane protease, serine 11e DESC1 | chr5 | inside |
| Tnfrsf19 | RELT tumor necrosis factor receptor | chr7 | inside |
| Tnni2 | troponin I, skeletal, fast 2 | chr7 | promoter |
| Toe1 | target of EGR1, member 1 (nuclear) | chr4 | divergent |
| Tox | thymus high mobility group box protein | chr4 | inside |
| Trim28 | tripartite motif protein 28, KAP-1, KRIP-1, Tif1b | chr7 | promoter |
| Trpd52l3 | tumor protein D52-like 3 | chr19 | promoter |
| Trpm6 | transient receptor potential cation channel, subfamily M, member 6 CHAK2 | chr19 | promoter |
| Tspan11 | tetraspanin 11 | chr6 | promoter |
| Tspan31 | tetraspanin 31, Sas, Tspan31 | chr10 | divergent |
| Ttll12 | tubulin tyrosine ligase-like family, member 12 | chr15 | promoter |
| Ttll6 | tubulin tyrosine ligase-like family, member 6 | chr11 | promoter |
| Ubxd3 | UBX domain containing 3 | chr4 | promoter |
| Uqcrh | ubiquinol-cytochrome c reductase hinge protein | chr4 | inside |
| Usp18 | ubiquitin specific peptidase 18 UBP43 | chr6 | promoter |
| Usp25 | ubiquitin specific peptidase 25 | chr16 | promoter |
| Utp18 | UTP18, small subunit (SSU) processome component, homolog (yeast), Wdr50 | chr11 | inside |
| V1rd6 | vomeronasal 1 receptor, D6 V3R6 | chr7 | promoter |
| Vangl1 | vang-like 1(van gogh, Drosophila), KITENIN, Lpp2, mStbm, stbm 2 | chr3 | inside |
| Vax1 | ventral anterior homeobox 1 | chr19 | promoter |
| Veph1 | ventricular zone expressed PH domain homolog 1 (zebrafish), Veph | chr3 | promoter |
| Vil2 | villin 2 cytovillin, ezrin, p81 NB ezrin is new symbol | chr17 | promoter |
| Vps25 | vacuolar protein sorting 25 (yeast) | chr11 | promoter |
| Wasf1 | WASP family 1,Scar, WAVE, WAVE-1 | chr10 | promoter |
| Wdr33 | WD repeat domain 33 | chr18 | promoter |
| Wnt3a | wingless-related MMTV integration site 3A | chr11 | inside |
| Wt1 | wilms tumor (Nephroblastoma) homolog | chr2 | promoter |
| Xlkd1 | lymphatic vessel endothelial hyaluronan receptor 1, lymphatic vessel endothelial HA receptor-1, Lyve-1 | chr7 | inside |
| Xpo6 | exportin 6, Ranbp20 | chr7 | inside |
| Zap70 | zeta-chain (TCR) associated protein kinase Srk, TZK, ZAP-70 | chr1 | promoter |
| Zbtb41 | zinc finger and BTB domain containing 41 homolog | chr1 | promoter |
| Zdhhc17 | zinc finger, DHHC domain containing 17, Hip14 | chr10 | inside |
| Zfp206 | zinc finger and SCAN domain containing 10 Zfp206, Zscan10 | chr17 | inside |
| Zfp27 | zinc finger protein 27 mkr-4 mszf76 | chr7 | promoter |
| Zfp276 | zinc finger protein (C2H2 type) 276 | chr8 | promoter |
| Zic2 | Zic finger protein of the cerebellum 2, GENA 29, Ku, odd-paired homolog | chr14 | inside |
| Zic5 | zinc finger protein of the cerebellum 5, odd-paired related, Opr | chr14 | divergent |
| Zw10 | ZW10 homolog (Drosophila), centromere/kinetochore protein MmZw10 | chr9 | promoter |
| Hoxc8 | Homeo box C8 | chr15 | promoter |
| Lef1 | Lymphoid Enhancer Binding Factor 1 | chr3 | inside |
| Sall1 | Spalt-like transcription factor 1 Msal-3 | chr8 | promoter |
| Sox1 | SRY-box containing gene 1 | chr8 | promoter |
