# Supplementary material for: Genomic Targets of Brachyury (T) in Differentiating Mouse Embryonic Stem Cells
Source: PLoS One. 2012 Mar 30;7(3):e33346. doi: 10.1371/journal.pone.0033346 (PMC3316570; doi:10.1371/journal.pone.0033346)
Supplement: Table S2 — Targets involved in key signalling pathways. (DOC) [file pone.0033346.s008.doc]

**Supplementary Table S2**

**Members of Key Signaling Pathways**

| **Target Gene** | **Key pathway** | **Role** | **Non-canonical & additional pathways** |
| --- | --- | --- | --- |
| Axin2/conductin | Wnt signaling pathway | Inhibitor of wnt signaling, associates APC/βcatenin/GSKβ3/DVL | Crosstalk TGFβ pathway (Smad 3)  Crosstalk MEKK1  SAPK stress pathway |
| Ctnnb1/β catenin | Wnt signaling pathway | LEF/TCF dependent transcriptional activation | A Component of adherens junctions |
| Dkk1/dickkopf homolog 1 | Wnt signaling pathway | Antagonist wnt signaling, anterior side embryo |  |
| Dvl3/dishevelled 3 | Wnt signaling pathway | Dvl1/3 <<< Dvl2 pool  Signaling canonical pathway sensitive to Dvl3 levels | JNK signaling  PCP pathway  Nodal pathway |
| Jup/Ctnng  plakoglobin/γ-catenin | Wnt signaling pathway | Associates APC and axin  Possible TCF/LEF transcriptional activity | A Component of adherens junctions and desmosomal plaques |
| Ppp2r2b | Wnt signaling pathway | Modulation of PP2A activity | Tight junctions |
| Wnt3a | Wnt signaling pathway | AP elongation, somite, tailbud formation | JNK signaling PCP pathway |
| Map2k5/MEK5 | MapK signaling pathway | *In vivo* activator of ERK5 |  |
| Map3k11/PTK1/MLK3 | MapK signaling pathway | Positive regulator JNK signaling | NF-κβ signaling  JNK pathway TNF signaling |
| Map3k2/MEKK2 | MapK signaling pathway | Activates MEK5  Required for T cell signaling | GnRH signaling pathway |
| Map3k3/MEKK3 | MapK signaling pathway | Activates MEK5 cardiovascular development | GnRH signaling pathway |
| Cdc42ep5/CEP5 | c-Jun N-Terminal Kinase (JNK) pathway | Regulation of cell shape | Rho GTPase |
| Junb | JNK pathway | Regulates erythroid cell survival, proliferation, and differentiation | Smad interacting protein |
| Syk | JNK pathway | Cell-cell adhesion via E cadherin & α catenin | NF-κβ signaling  B cell Receptor Signaling  T cell Receptor Signaling  Ras/MAPK signaling |
| BMP1 | TGFβ signaling pathway | Tolloid related |  |
| Fut 8 | TGFβ signaling pathway | Required for VEGFR2 expression | N-glycan biosynthesis  Keratin sulphate biosynthesis  Integrin mediated signaling |
| GDF5 | TGFβ signaling pathway | Embryonic limb morphogenesis | Cytokine-cytokine receptor interaction |
| BOC | Hedgehog Signaling pathway | Robo related, sonic hedgehog receptor  Required myogenesis |  |
| ERG | Fgf signaling | Regulation angiogenesis | Receptor signaling  B cell receptor signaling |
| FGF8 | Fgf signaling | L-R determinant, competence to respond to nodal signals | actin cytoskeleton signaling |
| Adam 19 | Integrin signaling | Heart development & dendritic cell marker |  |
| Adam 24 Testase 1 | Integrin signaling | Protease implicated in sperm function during epididymal maturation or fertilization |  |
| Rhou / WRCH1 | Integrin signaling | Wnt-1 responsive Cdc42 hom stimulates cells to reenter the cell cycle |  |
| Akap10 (D-AKAP2) | G-protein Coupled Receptor signaling | Localisation camp dep PKA , implicated heart rhythm regulation | AMP mediated signaling |
| Arhgef4 | G-protein Coupled Receptor signaling | cell migration & cell-cell adhesion | Rho guanine nucleotide exchange factor |
| Camk2a | G-protein Coupled Receptor signaling |  | B cell receptor signaling |
| Gnaq | G-protein Coupled Receptor signaling | Vascular smooth muscle contraction  cell morphogenesis | GnRH signaling pathway  Wnt pathway |
| Gnaz | G-protein Coupled Receptor signaling |  |  |
| Gnb2l1/Rack1 | G-protein Coupled Receptor signaling | Localized to proliferating endothelial cells |  |
| Ptk2b, Pyk2, Raftk | G-protein Coupled Receptor signaling | Cell adhesion  Activation EGFR | GnRH signaling pathway  Calcium signaling pathway |
| Lphn3/LEC3 | G-protein Coupled Receptor signaling | Ca^2+^independent receptor | neuropeptide signaling pathway |
| Rgs2 | G-protein Coupled Receptor signaling | Negative regulator of G coupled receptor signalling |  |
| Sstr3 | G-protein Coupled Receptor signaling | May activate G-proteins at tight junctions | Neuroactive ligand-receptor interaction |
| Bcl6 | B cell Receptor signaling | Repressor of transcription, memory B cell development |  |
| Chn2 | T cell Receptor signaling | Proliferation & migration SMC | Rac-specific GTPase activating proteins |
| Dpp4/CD26 | T cell Receptor signaling |  |  |
| Zap70 | T cell Receptor signaling | Like syk non receptor PK. Associates TCR zeta chain | NK-κβ signaling |
| IL6 | Cytokine/Jak/STAT pathway | First cytokine developing circulation osteoblasts to osteoclasts |  |
| Lif | Cytokine/Jak/STAT pathway | Down regulated on differentiation cell morphogenesis | Gastrulation is inhibited in mouse embryos overexpressing LIF |
| Lifr | Cytokine/Jak/STAT pathway | Down regulated on differentiation |  |
| Acly | Insulin Receptor signaling | Synthesis Acetyl CoA  May have additional tissue specific function development | TCA cycle CO_2_ fixation |
| TGFα | ErbB/HER signaling | Angiogenesis, cell proliferation |  |
| Pck2/PEPCK | PPAR signaling pathway |  | Adipocytokine signaling pathway |
| Gcnt2 | Sphingolipid Metabolism & N-Glycan Metabolism | Branching enzyme responsible conversion fetal to adult blood antigens |  |
| Neu3 | Sphingolipid Metabolism & N-Glycan Metabolism | skeletal muscle differentiation  Suppression of apoptosis  Mobilises membrane ruffles |  |
| St8sia3 | Sphingolipid Metabolism & N-Glycan Metabolism | Fetal blood formation & neuronal development |  |
| Hsd17b3 | Androgen & Estrogen Metabolism | Lipid biosynthesis |  |
| St6galnac1 | O-glycan Biosysnthesis | Decreases cell adhesion & increases cell migration |  |
| Pde2aPdpk1 | Purine Metabolism | Phosphodiesterase activity  Cell communication |  |
| Pde7b | Purine Metabolism | Phosphodiesterase activity |  |
| Pold3 | Purine & Pyrimidine Metabolism | DNA replication & repair subunit DNA polδ |  |
| Prune | Purine Metabolism | Pyrophosphatase activity |  |
| Nedd4l | Protein Ubiquitination pathway | ubiquitin protein-ligase activity |  |
| Sumo2 | Protein Ubiquitination pathway | Protein modification | NK-κβ signaling |
| Usp18 | Protein Ubiquitination pathway | Deubiquitinating enzyme |  |
| Usp25 | Protein Ubiquitination pathway | Deubiquitinating enzyme |  |
| Appbp1 | NEDD8 conjugation pathway | Neddylation | Apoptosis and cell cycle progression |
| Ephx1 | NFR2 Mediated Oxidative Stress Response | Protective enzyme |  |
| Gstm7 | NFR2 Mediated Oxidative Stress Response |  | Glutathione metabolism |
| Rab5b | Clathrin & Calveolar Mediated Endocytosis | Establishment of localization | small GTPase mediated signal transduction |
| Abcc2Mrp2 | RXR Activation | Membrane transport multidrug resistance | ABC Transport |
| Robo4 | Src Family Kinase Activation | Vascular -specific inhibits endothelial migration | Slit-Robo signaling |
| Cit | Protein serine/threonine kinase activity | Tissue specific neural tube regulator of cytokinesis | Rho interacting kinase |
| Dscr1l1 Rcan2 | Calcium mediated signaling | Regulation of calcineurin | calcineurin-NFAT pathway. |
