# Supplementary material for: Genomic Targets of Brachyury (T) in Differentiating Mouse Embryonic Stem Cells
Source: PLoS One. 2012 Mar 30;7(3):e33346. doi: 10.1371/journal.pone.0033346 (PMC3316570; doi:10.1371/journal.pone.0033346)
Supplement: Table S3 — Genes associated with germ cell development. (DOC) [file pone.0033346.s009.doc]

**Supplementary Table S3**

Germ Cell Associated Targets

| **Gene** | **Alt. Symbol** | **Name** |
| --- | --- | --- |
| 1700012A16Rik | MGI1919086 |  |
| 1700029I08Rik | AV259599, Actl9 | Actin-like 9 |
| Ooep | 2410146L05Rik,  Floped, Moep19 | Ooep, oocyte expressed protein homolog (dog) |
| 4930412F15Rik | MGI2441678 |  |
| 4930441O14Rik |  |  |
| 4930504O13Rik |  |  |
| 4930578I06Rik | AI429111 |  |
| Adam5 | tMDCII | A Disintegrin and metallopeptidase domain 5 |
| Adam19 | Mltnb | A Disintegrin and metallopeptidase domain 19 (meltrin beta) |
| Adam24 | Dtgn5 | A Disintegrin and metallopeptidase domain 24 (testase 1) |
| Asap1 | Ddef1 | Development and differentiation enhancing Asap1  ArfGAP with SH domain, ankyrin repeat and PH domain 1 |
| Cmtm2b | Cklfsf2b  1700013O04Rik | CKLF-like MARVEL transmembrane domain containing 2B homolog of human CKLFSF2 |
| Fam196b | Gm6041  EG574403 | Predicted gene, conserved  Family with sequence similarity 196, member B |
| Gsg1 |  | Germ cell-specific gene 1, germ cell associated 1 |
| Hsd17b3 | 17(beta) HSD type 3 | Hydroxysteroid (17-beta) dehydrogenase 3 |
| Id4 | Idb4 | Inhibitor of DNA binding 4, bHLHb27 |
| Morn2 | Mopt | MORN repeat containing 2 Mopt |
| Pfn4 | 2900024P18Rik | Profilin family, member 4 testis specific |
| Ttll6 | 633044416Rik | tubulin tyrosine ligase-like family, member 6 |
| Wdr33 | WDC146 | WD repeat domain 33, 1110001N06Rik, 2310011G05Rik, 2810021O11Rik, 8430413N20Rik |
| Wt1 | D630046I16Rik | Wilms tumor (Nephroblastoma) homolog |
| Zfp206 | Zscan10 | zinc finger and SCAN domain containing 10 |
