# Supplementary material for: Genomic Targets of Brachyury (T) in Differentiating Mouse Embryonic Stem Cells
Source: PLoS One. 2012 Mar 30;7(3):e33346. doi: 10.1371/journal.pone.0033346 (PMC3316570; doi:10.1371/journal.pone.0033346)
Supplement: Table S4 — Targets identified as transcription factors. (DOC) [file pone.0033346.s010.doc]

**Supplementary Table S4**

Transcription Factors Bound

| **Gene** | **Alt. Symbol** | **Name** |
| --- | --- | --- |
| Anxa7 | Anx7 | Annexin A7, synexin |
| Arid1a | BAF250a | AT rich interactive domain 1A (Swi1 like) |
| Atoh7 | Math5 | Atonal homolog 7 (*Drosophila*) |
| Bapx1 | Nkx-3.2 | NK3 homeobox 2 |
| Bcl6 | Bcl5 | B-cell leukemia/lymphoma 6 |
| C79407 |  | DNA binding protein |
| Cdk9 | PITALRE | Cyclin-dependent kinase 9 (CDC2-related kinase) |
| Cebpa |  | CCAAT/enhancer binding protein (C/EBP), alpha |
| Ctnnb1 | Catnb; Mesc | Catenin (cadherin associated protein), beta 1 |
| Ddef1 | Asap1 | Development and differentiation enhancing |
| Dlx5 |  | Distal-less homeobox 5 |
| Ebf1 | O/E-1, Olf1 | Early B-cell factor 1 |
| Ebf2 | D14Ggc1e, Mmot1, O/E-3 | Early B-cell factor 2 |
| Erg |  | Avian erythroblastosis virus E-26 (v-ets) oncogene related |
| Etv1 | ER81, Etsrp81 | Ets variant gene 1 |
| Fbxl10 | Jhdm1b, JEMMA | F-box and leucine-rich repeat protein 10, Jumonji domain, EMSY-interactor, methyltransferase motif |
| Fev | mPet-1, Pet1 | FEV (ETS oncogene family), Fifth Ewing Variant |
| Foxa2 | HNF3beta, Tcf3b | Forkhead box A2 |
| Foxe1 | Titf2 | Forkhead box E1 (thyroid transcription factor 2) |
| Foxi2 | B130055A05Rik | Forkhead box I2 |
| Foxn1 | Hfh11, whn | Forkhead box N1 |
| Hoxa13 | Hox-1.10 | Homeo box A13 |
| Hoxa3** |  | Homeo box A3 |
| Irx6 |  | Iroquois related homeobox 6 (*Drosophila*) |
| Jun B | AP-1 | Jun-B oncogene/AP-1 activator protein 1 |
| Lhx5 | Lim2 | LIM homeobox protein 5 |
| Lmx1b | LMX1.2 | LIM homeobox transcription factor 1 beta |
| Meis1 | C530044H18Rik | Meis homeobox 1 |
| Msgn1 |  | Mesogenin 1 |
| Nkx2-6 | Tix | NK2 transcription factor related, locus 6 Tinman |
| Pax 2 |  | Paired box gene 2 |
| Pax 3 |  | Paired box gene 3 |
| Pax 5 | EBB-1 | Paired box gene 5, B cell-specific activator protein |
| Pdx1 | IDX-1, Ipf1, Mody4, STF-1 | Pancreatic and duodenal homeobox 1 |
| Ppargc1a | Pgc-1alpha, Pgc1, Pgco1 | Peroxisome proliferative activated receptor, gamma, coactivator 1 alpha |
| Ppargc1b | ERRL1, PGC1 beta | Peroxisome proliferative activated receptor, gamma, coactivator 1 beta |
| Snai2 | Slug, Snail2 | Snail homolog 2 (*Drosophila*) |
| Snx26 | Tcgap | Sorting nexin 26 |
| Stra13 |  | Stimulated by retinoic acid 13 |
| Stra6 |  | Stimulated by retinoic acid gene 6 |
| Tcf19 | 5730403J10Rik | Transcription factor 19 |
| Tfg | Trk-fused gene | Trk-fused gene |
| Tox | 1700007F02Rik | Thymus high mobility group box protein |
| Trim28 | KAP-1, KRIP-1 | Tripartite motif protein 28 |
| Usp18 | UBP43 | Ubiquitin specific peptidase 18 |
| Usp25 |  | Ubiquitin specific peptidase 25 |
| Vax1 |  | Ventral anterior homeobox 1 |
| Wt1 | D630046I19Rik | Wilms tumor (Nephroblastoma) homolog |
| Zbt41 | 8430415N23Rik  9830132G07Rik | Zinc finger and BTB domain containing 41 homolog |
| Zfp206 | Zscan10 | Zinc finger and SCAN domain containing 10 Zfp206 |
| Zfp27 | mkr-4 mszf76 | Zinc finger protein 27 |
| Zfp276 | D8Ertd370e, D8Ertd377e | Zinc finger protein (C2H2 type) 276 |
| zic2 | GENA 29, Ku | Zinc finger protein of the cerebellum 2 odd-paired homolog |
| zic5 | Opr | Zinc finger protein of the cerebellum 5 odd-paired related |

** Agilent software miss-called HoxA2 as target, binding peak in 5’UTR HoxA3
