# Supplementary material for: Genomic Targets of Brachyury (T) in Differentiating Mouse Embryonic Stem Cells
Source: PLoS One. 2012 Mar 30;7(3):e33346. doi: 10.1371/journal.pone.0033346 (PMC3316570; doi:10.1371/journal.pone.0033346)
Supplement: Table S5 — Conservation of AC repeats. (DOC) [file pone.0033346.s011.doc]

**Supplementary Table S5**

**List of genes with AC repeats (with at least 8 nucleotides), their peak coordinates and sequence conservation analysis (presence of at least 8 nucleotides) in other species**

| **Gene** | **Peak Coordinates** | **Conservation** |
| --- | --- | --- |
| 1700029I08Rik | chr17:33034104-33034403 | R |
| 1700029I08Rik | chr17:33034240-33034539 | R |
| 4732495E13Rik | chr15:79229514-79229813 | R |
| 4930404H21Rik | chr7:133438288-133438587 | R |
| 4930556P03Rik | chr15:99303073-99303372 | - |
| AA407270 | chr9:71391930-71392229 | H |
| AA407270 | chr9:71391930-71392229 | H |
| Abcc2 | chr19:43832060-43833309 | R |
| Adar | chr3:89817795-89818142 | R |
| Ankrd13d | chr19:4271423-4271722 | - |
| Aqp11 | chr7:97614984-97615283 | - |
| Atp6v1d | chr12:79779006-79779305 | R |
| BC035295 | chr15:100324504-100324803 | R |
| BC048651 | chr6:29268348-29268647 | R |
| BC052496 | chr15:80395457-80395824 | - |
| Bcl6 | chr16:23906124-23906653 | R |
| Boc | chr16:44476590-44477179 | R, H |
| Car10 | chr11:92916538-92916837 | R, H |
| Carhsp1 | chr16:8591255-8591554 | - |
| Ccdc85a | chr11:28484974-28485273 | - |
| Cebpa | chr7:34824106-34824552 | R |
| Chn2 | chr6:54197731-54198030 | R |
| Chrnb1 | chr11:69610020-69610319 | - |
| Cit | chr5:116227765-116228064 | - |
| Cit | chr5:116227898-116228197 | - |
| Cml4 | chr6:85798316-85798615 | - |
| Cnga3 | chr1:37158481-37158780 | R |
| Cnn1 | chr9:21847791-21848090 | R |
| Coq2 | chr5:100918565-100918864 | - |
| Crygn | chr5:24265622-24265921 | - |
| D130058I21Rik | chr11:72232485-72232784 | R |
| Dkk1 | chr19:30614182-30615042 | - |
| Dpp4 | chr2:62213205-62213504 | R |
| Dvl3 | chr16:20431495-20431777 | R |
| Ebf2 | chr14:66183281-66183930 | R |
| Etv1 | chr12:39287680-39288047 | R |
| Fabp1 | chr6:71128736-71129035 | - |
| Fev | chr1:74824484-74824783 | R, H |
| Fgf8 | chr19:45798051-45798373 | R |
| Foxa2 | chr2:147741739-147742038 | - |
| Foxe1 | chr4:46361799-46362327 | R |
| Fyb | chr15:6524500-6524799 | R |
| Gdf5 | chr2:155634454-155634753 | H |
| Hcrtr1 | chr4:129642547-129643326 | R, H |
| Hnrpul1 | chr7:25461007-25461306 | - |
| Hoxa13 | chr6:52190959-52191258 | R |
| Hoxa2 | chr6:52102665-52103432 | R, H |
| Id4 | chr13:48270971-48271270 | - |
| Junb | chr8:87873059-87873358 | R, H |
| Kcnn4 | chr7:24077403-24077702 | R |
| Lama4 | chr10:38656104-38656934 | R |
| Lhfpl2 | chr13:95155518-95155817 | - |
| Lhx5/Lim2 | chr5:120694575-120694874 | R |
| Map2k5 | chr9:63180895-63181194 | R |
| Mapre2 | chr18:23942472-23942710 | - |
| Mbp | chr18:82610668-82610967 | R |
| Meis1 | chr11:18922898-18923197 | H |
| MGC117608 | chr6:12058209-12058508 | - |
| Morn2 | chr17:80196589-80196888 | - |
| Mov10 | chr3:104946714-104947013 | - |
| Mrpl14 | chr17:45150894-45151193 | R |
| Mttp | chr3:138067297-138067596 | R |
| Nat3 | chr8:70450322-70450621 | R |
| Nlrp4b | chr7:9561454-9561753 | R |
| Npffr2 | chr5:90600955-90601254 | R |
| Oas1f | chr5:121109912-121110211 | R |
| Olfr473 | chr7:107719324-107719623 | R |
| Olfr71 | chr4:43729710-43730009 | - |
| Olfr934 | chr9:38735851-38736150 | R |
| Otop2 | chr11:115124749-115125048 | R |
| Pax3 | chr1:78077797-78077993 | R |
| Pdzd9 | chr7:120469324-120469623 | R |
| Pelp1 | chr11:70227398-70228397 | R |
| Pigk | chr3:152650934-152651233 | R |
| Pitpnc1 | chr11:107287768-107288067 | R |
| Pogk | chr1:168247826-168248125 | H |
| Pold3 | chr7:99997329-99997628 | - |
| Ppm1m | chr9:106060544-106060843 | - |
| Pqlc3 | chr12:17027118-17027417 | R |
| Prune | chr3:95366332-95366631 | R |
| Psmd2 | chr16:20560174-20560473 | - |
| Ptn | chr6:36738757-36739401 | R, H |
| Rab17 | chr1:92802585-92802884 | - |
| Rab6b | chr9:102969066-102969365 | - |
| Rsl1 | chr13:67669189-67669488 | R |
| Rttn | chr18:89104706-89105203 | R |
| Rufy3 | chr5:89658618-89658917 | R, H |
| Sart2 | chr10:33899902-33900432 | R |
| Sart2 | chr10:33897285-33897822 | R, H |
| Schip1 | chr3:68658542-68658841 | R |
| Serpinb1c | chr13:32905891-32906190 | - |
| Sertad2 | chr11:20439814-20440113 | - |
| Slc41a3 | chr6:90566229-90566838 | - |
| Smarca2 | chr19:26669021-26669320 | - |
| St6galnac1 | chr11:116594420-116594719 | R |
| Syk | chr13:52604075-52604374 | R, H |
| Tcl1b2 | chr12:105548348-105548647 | R |
| Tiprl | chr1:167074926-167075225 | - |
| Tnfrsf19 | chr7:100736389-100736688 | R |
| Tnni2 | chr7:142250655-142250954 | - |
| Tox | chr4:6916154-6916453 | R |
| Trim28 | chr7:11923729-11924028 | - |
| Tspan31 | chr10:126478722-126478891 | - |
| Ttll6 | chr11:95947178-95947477 | R, H |
| Ubxd3 | chr4:138009399-138009698 | - |
| V1rd6 | chr7:5615400-5615699 | R |
| V1rg3 | chr7:11100370-11100974 | - |
| Vil2 / ezrin | chr17:6635815-6636274 | R |
| Wasf1 | chr10:40567697-40567996 | R |
| Zfp27 | chr7:29617531-29617830 | R |
| Zic2 | chr14:121611987-121612286 | R |

| **Key** | **Species** |
| --- | --- |
| - | Mouse |
| R | Rat |
| H | Human |
