# Supplementary material for: Genomic Targets of Brachyury (T) in Differentiating Mouse Embryonic Stem Cells
Source: PLoS One. 2012 Mar 30;7(3):e33346. doi: 10.1371/journal.pone.0033346 (PMC3316570; doi:10.1371/journal.pone.0033346)
Supplement: Table S6 — Quantitative PCR primers. (DOC) [file pone.0033346.s012.doc]

**Supplementary Table S6**

PCR primers used for quantitative RT-PCR analysis

| **Primer** | **Type** | **Sequence 5’ → 3’** | **Amplicon bp** |
| --- | --- | --- | --- |
| βactinF | Endogenous control | AGGTCATCACTATTGGCAACGA | 117 |
| βactinR | Endogenous control | CACTTCATGATGGAATTGAATGTAGTT |  |
| BrachyuryF | Primitive streak | GCTTCAAGGAGCTAACTAACGAG | 117 |
| BrachyuryR | Primitive streak | CCAGCAAGAAAGAGTACATGGC |  |
| Axin2F | Wnt Target gene | TGACTCTCCTTCCAGATCCCA | 105 |
| Axin2R | Wnt Target gene | TGCCCACACTAGGCTGACA |  |
| Ctnnb1F | Wnt Target gene | ATGGAGCCGGACAGAAAAGC | 108 |
| Ctnnb1R | Wnt Target gene | CTTGCCACTCAGGGAAGGA |  |
| Dkk1F | Wnt Target gene | CTCATCAATTCCAACGCGATCA | 105 |
| Dkk1R | Wnt Target gene | GCCCTCATAGAGAACTCCCG |  |
| Dvl3F | Wnt Target gene | GTCACCTTGGCGGACTTTAAG | 128 |
| Dvl3R | Wnt Target gene | AAGCAGGGTAGCTTGGCATTG |  |
| JupF | Wnt Target gene | TGGCAACAGACATACACCTACG | 135 |
| JupR | Wnt Target gene | GGTGGTAGTCTTCTTGAGTGTG |  |
| Wnt3a F | Wnt Target gene | ctcctctcggatacctcttagtg | 186 |
| Wnt3a R | Wnt Target gene | gcatgatctccacgtagttcctg |  |
| Bapx1F | Target gene | TCCAGGCGATCCTCAACAAGA | 248 |
| Bapx1R | Target gene | GGCTGAGTCTGAGTCCCAAC |  |
| ERGF | Target gene | ACCTCACCCCTCAGTCCAAA | 105 |
| ERGR | Target gene | TGGTCGGTCCCAGGATCTG |  |
| FevF | Target gene | ACGCCTACCGCTTTGACTTC | 186 |
| FevR | Target gene | AAGCTGCCATCAAGTTGAGTT |  |
| Foxa2F | Target gene | TAG CGG AGG CAA GAA GAC C | 150 |
| Foxa2R | Target gene | CTT AGG CCA CCT CGC TTG T |  |
| Foxe1F | Target gene | ATCGCGCTCATCGCTATGG | 108 |
| Foxe1R | Target gene | GGGGTTGTCGCGGTAGAAC |  |
| Hoxa2F | Target gene | TACGAATTTGAGCGAGAGATTGG | 116 |
| Hoxa2R | Target gene | GTCGAGGTCTTGATTGATGAACT |  |
| Hoxa3F | Target gene | TCAGCGATCTACGGTGGCTA | 250 |
| Hoxa3R | Target gene | GAGGCAAAGGTGGTTCACCC |  |
| Meis1F | Target gene | GCAAAGTATGCCAGGGGAGTA | 235 |
| Meis1R | Target gene | TCCTGTGTTAAGAACCGAGGG |  |
| Msgn1F | Target gene | CTTCTGACACCGCTGGTCTG | 188 |
| Msgn1R | Target gene | GTGACTGCCGTAGCCATCG |  |
| Pax3F | Target gene | GGGCAGAATTACCCACGCA | 177 |
| Pax3R | Target gene | CTGGCGAGAAATGACGCAA |  |
| Nkx2.6F | Target gene | gcattcctggtccctacaaa | 78 |
| Nkx2.6R | Target gene | agctagcgtcgtagggagtg |  |
| snai2F/Slug | Target gene | TGGTCAAGAAACATTTCAACGCC | 131 |
| snai2R/Slug | Target gene | GGTGAGGATCTCTGGTTTTGGTA |  |
