# Supplementary material for: Genomic Targets of Brachyury (T) in Differentiating Mouse Embryonic Stem Cells
Source: PLoS One. 2012 Mar 30;7(3):e33346. doi: 10.1371/journal.pone.0033346 (PMC3316570; doi:10.1371/journal.pone.0033346)
Supplement: Table S7 — In situ hybridisation, T box, and electrophoretic mobility shift assay primers. (DOC) [file pone.0033346.s013.doc]

**Supplementary Table S7**

**Mouse *in situ***, T-box and EMSA primers

| **Primer** | **Sequence 5’ → 3’** |
| --- | --- |
| Wnt3a Bam F | agggatccatggctcctctcggatac |
| Wnt3a Xba R | CGTCTAGACTACTTGCAGGTGTGCA |
| T BamH1 F | AGGGATCCATGAGCTCGCCGGGCACAGAGA |
| T Box Xho1 R | ACCGCTCGAGGGTGCCAGCACCAGGA |
| Sequencing Primer pGEX 5’ F | GGGCTGGCAAGCCACGTTTGGTG |
| Sequencing Primer pGEX 3’ R | CCGGGAGCTGCATGTGTCAGAGG |
| Motif 1 F BamH1 end | AGATCTCGGTTT**TCACACCT**ATAGACCG**GATCC** |
| Motif 1 R Bgl II end | GGATCCGGTCTAT**AGGTGTGA**AAACCGA**GATCT** |
| Mutated Motif 1F | AGATCTCGGTTT**GCACGCTT**ATAGACCG**GATCC** |
| Mutated Motif 1R | GGATCCGGTCTAT**AAGCGTGC**AAACCGA**GATCT** |
| Motif 2 F BamH1 end | AGATCTCAA**ACACACACACCT**AGA**GATCC** |
| Motif 2 R Bgl II end | GGATCTCTAG**GTGTGTGTGT**TTGA**GATCT** |
| Mutated Motif 2F | AGATCTCAA**ACTCGCACTCCT**AGAGATCC |
| Mutated Motif 2R | GGATCTCTAG**GAGTGCGAGT**TTGAGATCT |
| Motif 3 F Bgl II | AGATCTCGGTT**TCACACCT**ATAGACCT**ACACACACACCT**AGA**GATCC** |
| Motif 3 R BamH1 | GGATCTCT**AGGTGTGTGTGT**AGGTCTAT**AGGTGTGA**AACCGA**GATCT** |
| Mutated 3 F T site intact (AC)_n_ altered | AGATCTCGGTT**TCACACCT**ATAGACCT**ACTCGCACTCCT**AGAGATCC |
| Mutated 3 R T site intact (AC)_n_ altered | GGATCTCT**AGGAGTGCGAGT**AGGTCTAT**AGGTGTGA**AACCGAGATCT |
| Mutated 3 F T site altered (AC)_n_ intact | AGATCTCGGTT**GCACGCTT**ATAGACCT**ACACACACACCT**AGAGATCC |
| Mutated 3 R T site altered (AC)_n_ intact | GGATCTCT**AGGTGTGTGTGT**AGGTCTAT**AAGCGTGC**AACCGAGATCT |

The motifs investigated in EMSA experiments are highlighted in **bold**. Nucleotides in **blue bold** indicate mutated versions of these motifs. Nucleotides in **red bold** were included in oligonucleotides designed for unlabelled competition experiments and were absent from oligonucleotides intended for end labelling.
