# Supplementary material for: Genomic Targets of Brachyury (T) in Differentiating Mouse Embryonic Stem Cells
Source: PLoS One. 2012 Mar 30;7(3):e33346. doi: 10.1371/journal.pone.0033346 (PMC3316570; doi:10.1371/journal.pone.0033346)
Supplement: Table S8 — Genomic quantitative PCR primers. (DOC) [file pone.0033346.s014.doc]

**Supplementary Table S8**

**PCR primers used for genomic quantitative PCR**

MOUSE

| **Primer** | **Sequence 5’ → 3’** | **Amplicon bp** |
| --- | --- | --- |
| **Bound genes** |  |  |
| Axin2 F | TGGAAAGGAATTCGAAGGTG | 176 |
| Axin2 R | CTTCCTTGCACACTCATGGA |  |
| Foxe1 F | CACCCAGTGCTCACTGAAGA | 144 |
| Foxe1 R | CACGGTGAAGCCAGTACCTT |  |
| Mapre2 F | GCCACTGTGCACTCTGCTTA | 176 |
| Mapre2 R | CAGTTTCTTGCCCATCAGGT |  |
| Nkx2.6 F | AGAAGGGGCAAACAAGGAAT | 184 |
| Nkx2.6 R | GGCTGGTTGTGCCAAACTAT |  |
| Pax3 F | AATCTCCCTCCCTTGCAAAT | 199 |
| Pax3 R | CACTCCCTAGCCAGCAGAAC |  |
| Rttn F | GCTGGAGGTTGGGAAAAGAT | 158 |
| Rttn R | CTTCAAAAACAGGGCTGCAT |  |
| Vangogh F | TATGGTTGGGGCTTTGATGT | 148 |
| Vangogh R | GCAACTGCATCCCTGAAAAT |  |
| **Published Target** |  |  |
| Nanog 5’F | GCTTGAACCAGCCAGTTCTC | 200 |
| Nanog 5’R | TTCCTCTCCCTCAGCTACCA |  |
| **Negative Regions/Genes** |  |  |
| Nanog 3’ F | CACCCACCCATGCTAGTCTT | 150 |
| Nanog 3’ R | ACCCTCAAACTCCTGGTCCT |  |
| 1700010C24Rik F | GCTTTGCCACAGCAGACATA | 128 |
| 1700010C24Rik R | AGCCAACAAGGTGGACATTC |  |
| β Actin F | GGGAATACTCTGGGCTCTCC | 132 |
| β Actin R | CCCTGGCCTTGTATTTCTCA |  |

HUMAN

| **Primer** | **Sequence 5’ → 3’** | **Amplicon bp** |
| --- | --- | --- |
| **Bound genes** |  |  |
| AXIN2 F | AGCCCTAACCCCTGACCCCC | 105 |
| AXIN2 R | TGTGCAGCCGGGGAGGATCT |  |
| FGF8 F | AGCGATCAGTGGCATCGCGG | 128 |
| FGF8 R | TGGCAGGAGGAGCGGGAGAC |  |
| JUP F | CCCCTAAGCCGACCAGCGGA | 108 |
| JUP R | GGGAAGCAGAGAGGGCCGGA |  |
| WNT3A F | TTGGCCACACAGGGAAGCGG | 82 |
| WNT3A R | GTGGGGATGGGGGAGGGGTC |  |
| **Negative Region/Gene** |  |  |
| NCAPD2 F | gagcctttccacaccatcat | 109 |
| NCAPD2 R | gccgaggtggaattcaaata |  |
